# Design principles for selective polarization of PAR proteins by cortical flows

**DOI:** 10.1083/jcb.202209111

**Published:** 2023-06-02

**Authors:** Rukshala Illukkumbura, Nisha Hirani, Joana Borrego-Pinto, Tom Bland, KangBo Ng, Lars Hubatsch, Jessica McQuade, Robert G. Endres, Nathan W. Goehring

**Affiliations:** 1 The Francis Crick Institute, London, UK; 2 Institute for the Physics of Living Systems, University College London, London, UK; 3 Department of Life Sciences, Imperial College London, London, UK

## Abstract

Clustering of membrane-associated molecules is thought to promote interactions with the actomyosin cortex, enabling size-dependent transport by actin flows. Consistent with this model, in the *Caenorhabditis elegans* zygote, efficient anterior segregation of the polarity protein PAR-3 requires oligomerization. However, through direct assessment of local coupling between motion of PAR proteins and the underlying cortex, we find no links between PAR-3 oligomer size and the degree of coupling. Indeed, both anterior and posterior PAR proteins experience similar advection velocities, at least over short distances. Consequently, differential cortex engagement cannot account for selectivity of PAR protein segregation by cortical flows. Combining experiment and theory, we demonstrate that a key determinant of differential segregation of PAR proteins by cortical flow is the stability of membrane association, which is enhanced by clustering and enables transport across cellular length scales. Thus, modulation of membrane binding dynamics allows cells to achieve selective transport by cortical flows despite widespread coupling between membrane-associated molecules and the cell cortex.

## Teaser

Illukkumbura et al. reveal that cluster-dependent tuning of membrane binding rather than size-dependent coupling to the actomyosin cortex enables differential transport of PAR proteins by cortical flows in the *C. elegans* zygote.

## Introduction

Over half a century ago, it was observed that crosslinking of antigens on the surface of immune cells could induce their coalescence into a domain at the cell rear (Raff et al., 1970). This process of “capping” results from the rearward transport of cross-linked antigens by retrograde cortical actin flows linked to cell motility (Bray and White, 1988; Taylor et al., 1971). However, in the years since there has been persistent debate over precisely what membrane-associated components are flowing together with the actin cortex, leaving unclear the principles that define whether a particular molecule will be subject to long range transport by cortical flows (Sheetz et al., 1989; Holifield et al., 1990; Jacobson and Kapustina, 2019; Bretscher, 1996; Illukkumbura et al., 2020).

Polarization of the *Caenorhabditis elegans* zygote initially proceeds similarly to the process of antigen capping. Here, cortical actomyosin flows away from what will become the zygote posterior pole (Munro et al., 2004). In doing so, cortical flows segregate a conserved set of peripheral membrane-associated polarity proteins, PAR-3, PAR-6, and PKC-3 (aPKC) into a cap at what will become the anterior pole (Munro et al., 2004; Goehring et al., 2011b). As these PAR proteins are segregated, they are replaced on the posterior plasma membrane by a second set of PAR proteins (PAR-1, PAR-2, LGL-1, and CHIN-1; Cuenca et al., 2003; Goehring, 2014; Rose and Gönczy, 2014). Once flows cease, mutually antagonistic interactions between these anterior (aPAR) and posterior (pPAR) proteins maintain the resulting polarity domains independently of the actin cortex (Cuenca et al., 2003; Goehring et al., 2011a; Goehring et al., 2011b; Hao et al., 2006; Hoege et al., 2010; Kumfer et al., 2010; Lang and Munro, 2017).

Like capping, segregation of aPAR proteins depends critically on plasma-membrane associated clusters, in this case of PAR-3. PAR-3 cluster motion is tightly coupled to the actin cortex and disruption of a conserved N-terminal oligomerization domain prevents polarization (Dickinson et al., 2017; Li et al., 2010; Munro et al., 2004; Rodriguez et al., 2017; Wang et al., 2017). Because the membrane association of PAR-6 and PKC-3 is tightly linked to interactions with PAR-3, under normal conditions all three co-segregate (Beers and Kemphues, 2006; Rodriguez et al., 2017). However, when allowed to load onto the membrane independently of PAR-3, PAR-6, and PKC-3 fail to segregate (Rodriguez et al., 2017). Thus, the question arises: what is special about PAR-3 clusters that allows them to be so efficiently transported across length scales reaching tens of microns?

A key requirement for transport of molecules by cortical flow (advection) is that their motion be entrained by or coupled to that of the underlying actomyosin cortex. Variation in this coupling to the actin cortex, as is seen among different molecules that make up focal adhesions (Hu et al., 2007), could therefore provide one mechanism to achieve selective transport. It is intriguing therefore that PAR proteins are not generally known to be associated with the actin cortex—they diffuse laterally in the membrane and appear distinct from molecules that are directly bound by the actin cortical network (Costache et al., 2022; Goehring et al., 2011a). In addition, the dynamics of PAR-2 and PAR-6 behavior at the membrane is only minimally affected by actin depolymerization (Goehring et al., 2011a), suggesting any interactions must be weak if they exist. However, the exception to this behavior is PAR-3. Specifically, the mobility of PAR-3 clusters appears to be restricted by the actin cortex in embryos (Sailer et al., 2015). Thus, an attractive model for explaining cluster-dependent transport is that clustering results in an increase in effective friction with the cortex arising from size-dependent binding or corralling (Zmurchok and Holmes, 2022), similar to what has been proposed at the immunological synapse (Hartman et al., 2009). In such a model, clustering would effectively tune the physical coupling of molecules to the actomyosin cortex, allowing molecules to selectively “sense flows.” Consistent with such a model, recent work has postulated a strict size threshold for directional transport by cortical flows (Chang and Dickinson, 2022).

At the same time, clustering of molecules could also serve to reduce diffusivity and enhance membrane association via avidity effects, both of which would also be expected to enhance the efficiency of transport by cortical flow. Indeed, Chang and Dickinson (2022) have suggested that differences in segregation of oligomers are more likely to be due to alterations in membrane binding and diffusivity rather than differential coupling to the cortex. Consequently, resolving how molecules can be selectively transported by cortical actomyosin flows requires that we directly assess the relative contributions of advection, diffusion, and membrane association, a task which is particularly challenging for molecules that are not obviously clustered or that may diffuse extensively at the membrane, which limits one’s ability to detect advective motion directly.

Here, we develop a workflow to assess the mobility of distinct pools of PAR proteins including clustered and non-clustered variants based on single particle tracking in live embryos. Surprisingly, our data are inconsistent with models of size- or cluster-dependent changes in coupling to cortical flows. Instead, we find that all PAR proteins couple efficiently to cortical flows, with their segregation in the zygote being strongly influenced by the strength of membrane binding, which increases the timescale over which cortical flows advect PAR proteins. Consistent with this paradigm, stabilizing membrane binding could both partially rescue segregation of oligomerization-defective PAR-3 and induce anterior segregation of the normally posterior protein PAR-2, effectively inverting its normal polarity. Thus, oligomerization-dependent tuning of membrane association represents a key design principle for achieving selective segregation by cortical flows in polarized cells.

## Results

### Coupling of PAR-3 to actomyosin cortical flow is independent of oligomeric state and diffusivity

**Figure 1.**
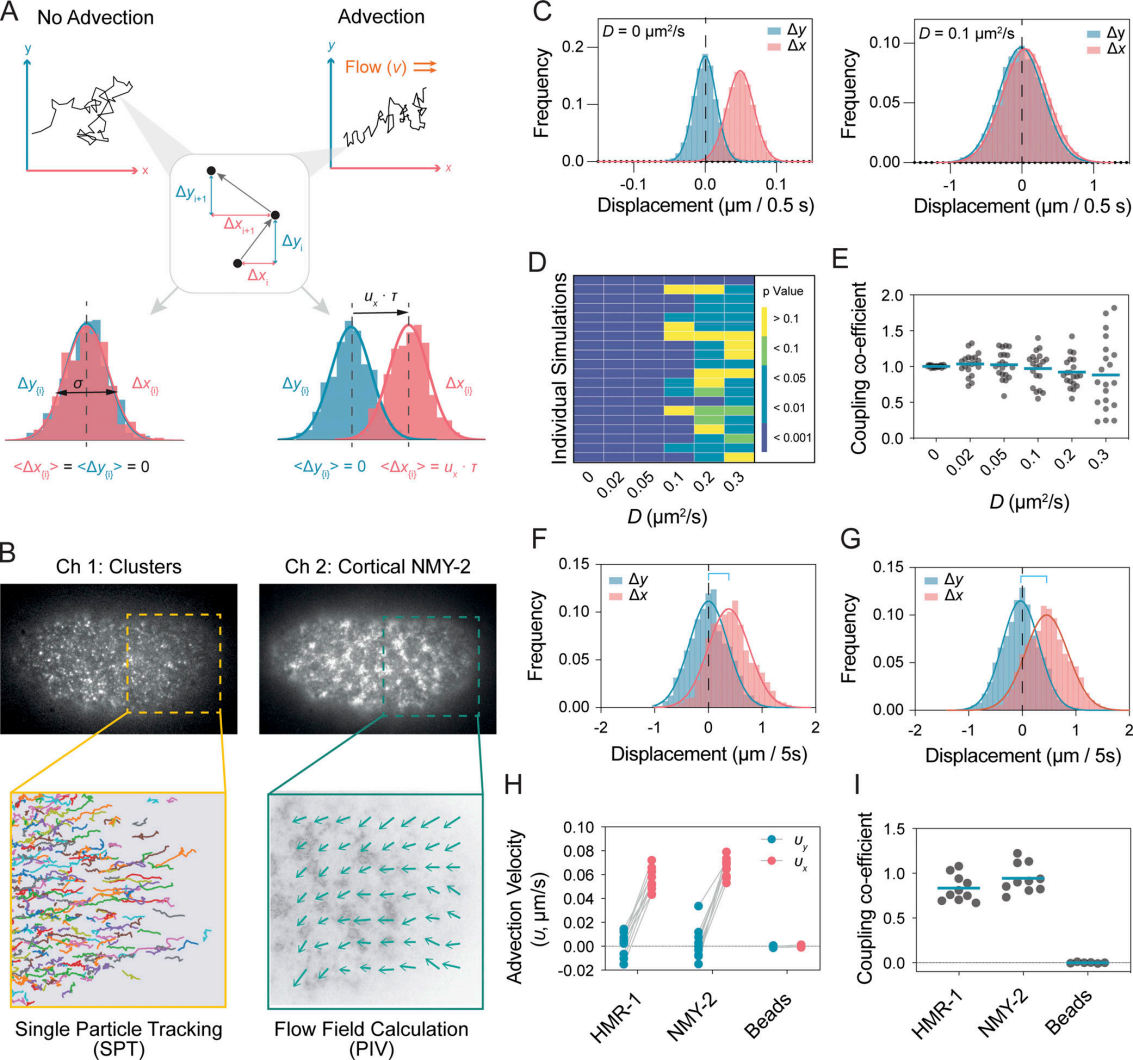
**Extracting advection and diffusion parameters from single particle trajectories. (A)** Schematic for decomposing trajectories into advective and diffusive components. Particle displacements are projected onto axes x and y defined as parallel and perpendicular to the local flow vector, respectively. In principle, these should take the form of a Gaussian. Drift is defined by the shift of the mean population displacement (<Δ*x*>, <Δ*y*>), along the relevant axis, where we expect <Δ*y*> ∼ 0. Advection velocity (*υ*_*x*_) is then given by mean displacement <Δ*x*>, divided by the time lag, τ, with the cortex coupling coefficient given by ratio of advection velocity (*υ*_*x*_) to local flow velocity $\left(cc=\upsilon_{x}/\nu \right).$ **(B)** Schematic for extraction of particle motion (PAR-3 clusters shown) and local flow field. Two-channel image series were captured for cortical NMY-2 and the molecule of interest using a HILO imaging regime. The resulting image series were subject to either a Python-based particle tracking scheme or particle image velocimetry (PIVLab, Matlab). Particle displacements for a given *τ* were then projected onto the relevant x- and y-axes defined by the local flow vector. Note, positive movement on the x-axis generally reflects motion toward the anterior. **(C)** Distribution of displacements in x and y for simulations of varying *D* for τ = 0.5 s. **(D)** Reliability of detection of drift as a function of *D*. Significance of difference between <Δ*x*> and <Δ*y*> calculated using 1,000 random displacements (τ = 0.5 s). Results shown for 20 independent simulations. Student’s *t* test, unpaired, two-tailed. **(E)** Mean *cc* ∼ 1.0 is obtained for all *D*, though error increases with *D*. Each point indicates *cc* measured from 1,000 random displacements from a single simulated dataset in D, with mean indicated. **(F and G)** Example of the distribution of displacements for NMY-2 (F) and HMR-1 (G) for single embryos (τ = 5 s, *n* = 1,000 randomly selected steps). In both cases, there is a characteristic drift component along the flow axis (x). Displacements parallel (red) and orthogonal (blue) to the local flow axis are shown. **(H and I)** Fit values for advection velocity for displacements parallel (*υ*_*x*_, red) and orthogonal (*υ*_*y*_, blue) to the flow axis (H) and coupling coefficients (I) shown for NMY-2 and HMR-1, as well as beads immobilized to the exterior of the eggshell. Lines in H connect paired data points from single embryos. Mean values for individual embryos shown in H and I, along with mean of all embryos in I.

To directly quantify the advection velocity of individual molecules, we developed a single particle workflow that allowed us to measure particle displacements relative to the local actomyosin flow field (Fig. 1 A). Actomyosin generally flows from posterior to anterior. However, to account for local variations in the flow vector, we simultaneously imaged the molecule of interest along with cortical actomyosin to obtain the local flow vector (Fig. 1 B). Displacements of the molecule of interest were then projected onto the local flow vector. From the parallel component, we could obtain an effective advection velocity, *υ*, that is aligned to the underlying NMY-2 flow-field. The ratio of *υ* to the velocity of the underlying actomyosin flow, *v*, defines a coupling coefficient, *cc*, where *cc* = *υ*/*ν*. The coupling coefficient reflects the degree to which the local velocity of the actin cortex is transduced to the molecule of interest, with *cc* = 1 indicating perfect coupling to the cortex and *cc* = 0 indicating no coupling. While this approach can in principle be used to analyze the motion of a single particle, in practice, imaging and tracking constraints, including limits on track length imposed by photobleaching, mean that steps from a population of particles often must be pooled to obtain sufficient data for fitting.

Simulated datasets suggest that under ideal circumstances and for reasonable numbers of embryo replicates (∼10), we should be able to detect advection for effective diffusivities of up to ∼0.3 µm^2^/s (Fig. 1, C–E). Given the diffusivities measured for PAR proteins are generally well below 0.2 µm^2^/s, the accuracy of our methodology is likely to be sufficient for our purposes (Arata et al., 2016; Goehring et al., 2011a; Gross et al., 2019; Hubatsch et al., 2019; Robin et al., 2014). To validate this workflow, we analyzed non-muscle myosin (NMY-2) and non-junctional E-cadherin (HMR-1), two molecules that are known to associate with the actin cortex and are advected by cortical flows in the zygote (Munro et al., 2004; Padmanabhan et al., 2017). As a negative control, we also analyzed beads that were associated with the exterior of the eggshell and thus physically isolated from flows. As expected, both HMR-1 and NMY-2 exhibited coupling coefficients near 1, while beads exhibited a coupling coefficient of zero (Fig. 1, F–I).

**Figure 2.**
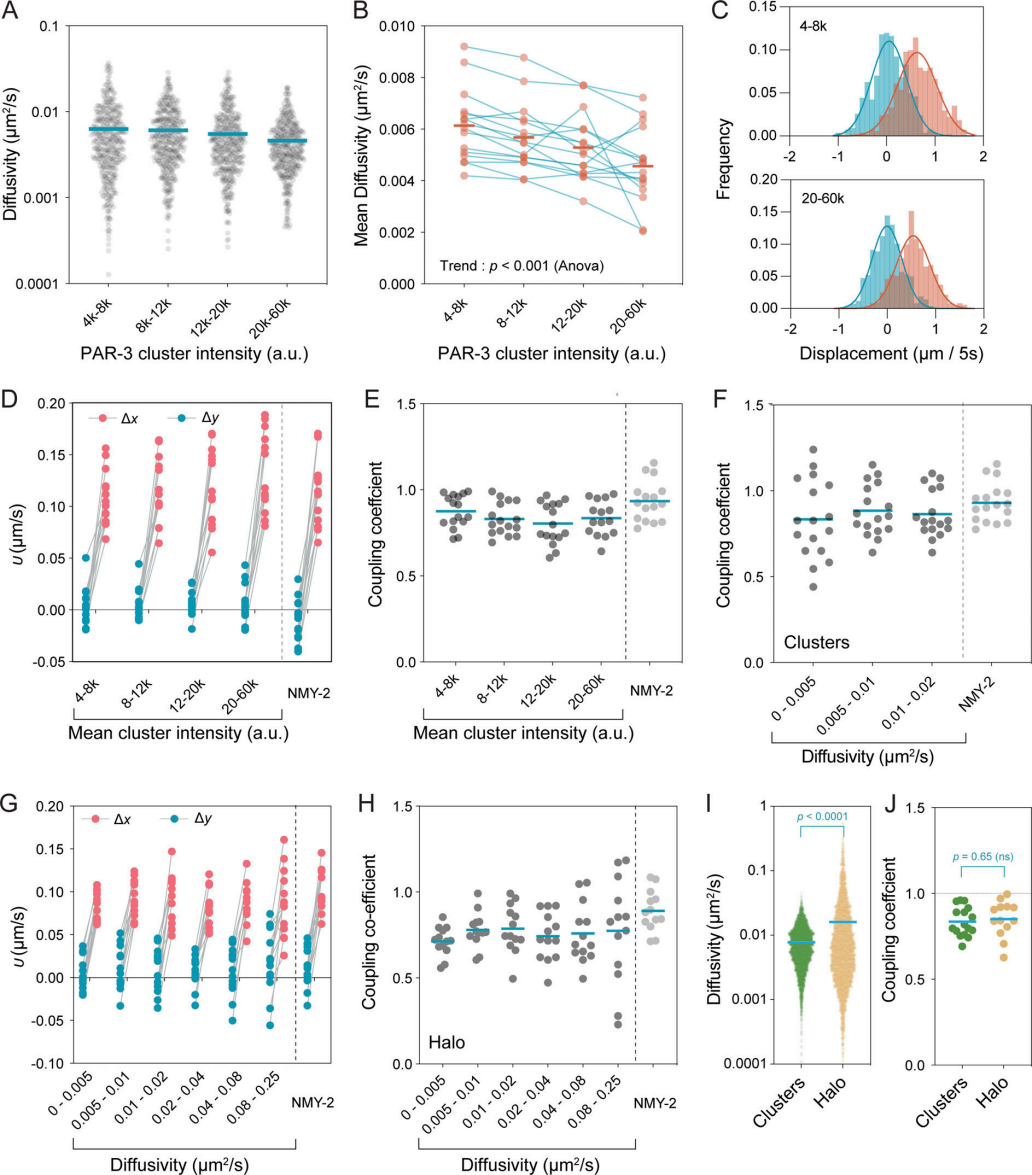
**Advection of PAR-3 is independent of cluster size. (A and B)** Cluster size only modestly affects diffusivity. **(A)** Distribution of diffusivities for 400 random clusters selected from all particles (16 embryos) for each of the indicated intensity bins (τ = 2 s). Mean values indicated. **(B)** Plot of mean cluster diffusivity per embryo across cluster size bins reveals a general trend of decreasing diffusivity with cluster size but note that clusters are generally very slowly diffusing in all bins. Mean values across all embryos indicated by bars. **(C)** The distribution of PAR-3 cluster displacements is shifted along the flow-parallel axis. Data shown for all clusters from a single embryo, τ = 5 s, *n* (steps) = 542, 1,033, 1,196, 844. **(D)** Fit values for advection velocity parallel (*υ*_*x*_, red) and perpendicular (*υ*_*y*_, blue) relative to the flow axis are similar for differently sized clusters. Lines connect paired values from single embryos. **(E and F)** Coupling coefficients are independent of cluster intensity, diffusivity. Coupling coefficients were fit from particles binned by indicated intensity bands (E) or diffusivity (F) on a per-embryo basis. Mean indicated. **(G)** Measured parallel (*υ*_*x*_, red) and perpendicular (*υ*_*y*_, blue) advection velocities (τ = 0.5 s) for single Halo-tagged molecules binned by diffusivity (τ = 0.2 s). **(H)** Coupling coefficients derived from Halo::PAR-3 single-molecule tracking are independent of diffusivity. Coupling coefficients were fit from molecules binned by indicated diffusivity on a per embryo basis. Mean indicated. **(I)** Distribution of effective diffusivities for PAR-3 clusters (green, *n* = 5,644, 16 embryos) and single JF-549 labeled molecules (yellow, *n* = 4,500, 12 embryos). For the purposes of this comparison, diffusivity was calculated using τ = 1 s in both cases. (Kolmogorov–Smirnov given non-normal distributions). **(J)** Coupling coefficients between clusters and Halo particles were not significantly different (unpaired *t*-test, two-tailed).

To directly assess whether there are size-dependent changes in how PAR-3 clusters couple to cortical flows, we tracked individual PAR-3 clusters during the polarity establishment phase. Taking mean cluster intensity as a proxy for size, we find that diffusivity decreased with cluster size. The observed changes were small, though we cannot rule out that measurement accuracy or embryo motion may mask differences among these very slowly diffusing particles (Fig. 2, A and B). Overall, cluster displacements exhibited Gaussian distributions consistent with lateral diffusion in the membrane, but with a clear shift in the mean displacement parallel to the flow vector, *x* (Fig. 2, C and D). Strikingly, fitting revealed nearly identical coupling coefficients across all size bins that were only modestly reduced relative to the NMY-2 reference (Fig. 2 E). Thus, at least for the range of cluster sizes that we could observe and track, coupling to cortical flows was insensitive to cluster size. We also re-binned particles by diffusivity to determine whether slower particles couple better to the cortex, reasoning that reduced diffusivity could reflect stronger corralling by the cortex. However, again we found that coupling coefficients were effectively independent of diffusivity (Fig. 2 F).

A caveat to these results is that imaging conditions optimized for clusters may fail to detect smaller, faster-diffusing species. Therefore, to better capture the full population and assess coupling to cortical flows as a function of diffusivity, we turned to a single-molecule labeling regime in which sub-stoichiometric labeling of endogenously Halo-tagged proteins was used to achieve a sparse set of well-separated trajectories that should better sample the overall population. After binning particles by diffusivity, we again measured the distribution of displacements. Strikingly, the coupling of particles to flows for even the fastest diffusing bins remained high and effectively unchanged compared to slower diffusing bins (Fig. 2, G and H). Note that Halo labeling was capable of revealing a faster diffusing population (Fig. 2 I), yet we saw no difference in coupling coefficient between GFP::PAR-3 clusters and Halo:PAR-3 molecules (Fig. 2 J).

**Figure 3.**
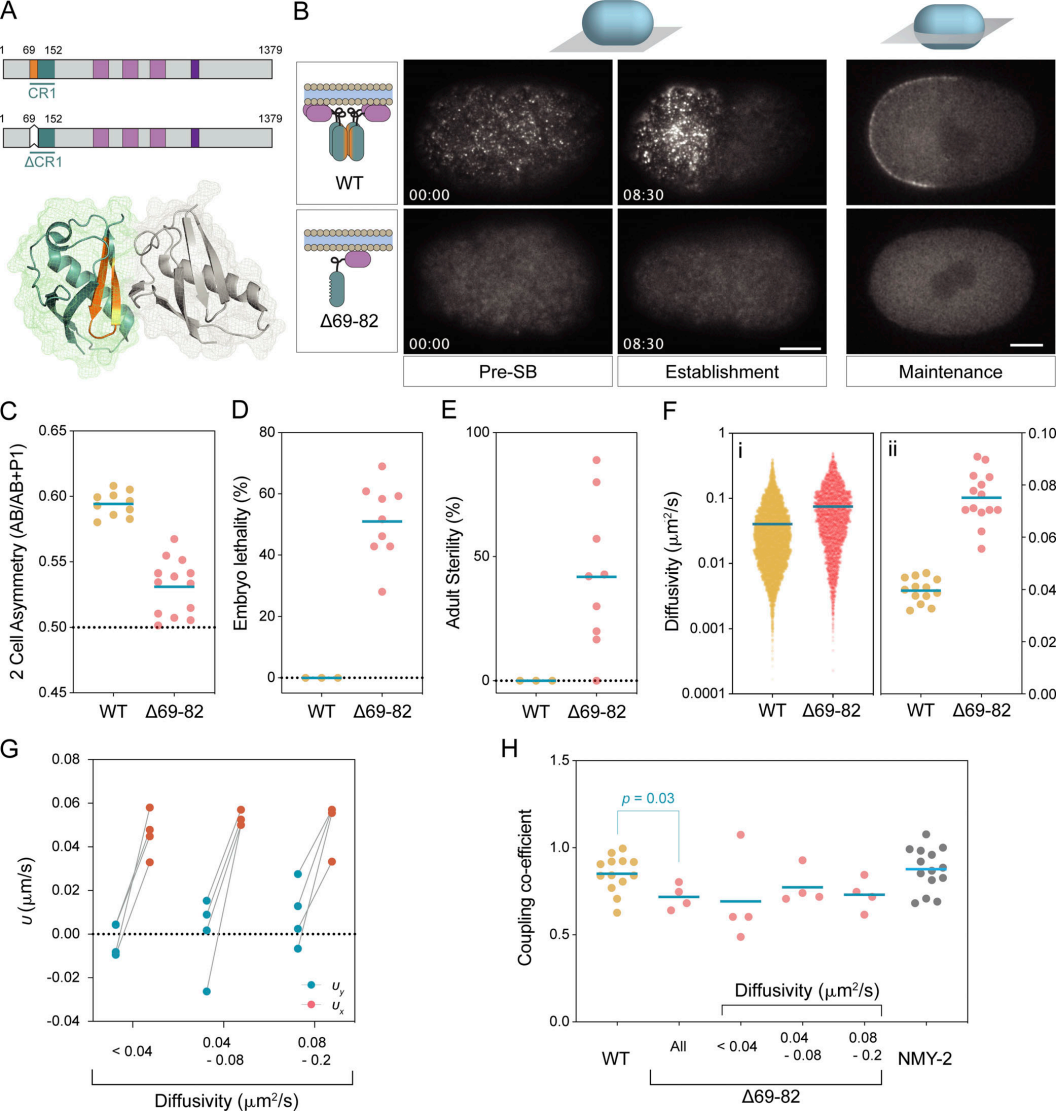
**Advective velocity of PAR-3 is independent of clustering. (A)** Schematic of full-length PAR-3 and PAR-3(∆69–82) and a crystal structure of the PAR-3 CR1 domain dimer (rat PAR3-NTD, PDB accession no. 4I6P) are shown. Individual CR1 molecules are shown in gray and blue. Amino acids 69–82 (orange) are located at the oligomerization interface and color-coded in the schematics. **(B)** PAR-3(∆69–82) does not accumulate on the anterior plasma membrane. Embryos expressing mNG fusions to either PAR-3(WT) or PAR-3(∆69–82) are shown in a cortical plane by HILO microscopy (Pre-Symmetry-Breaking, Establishment) or at midplane (Maintenance). Note general lack of signal at the plasma membrane, consistent with the requirement of oligomerization for stable membrane association. Scale bar, 10 µm. Time mm::ss relative to symmetry breaking. **(C–E)** PAR-3(∆69–82) leads to loss of P0 division asymmetry, embryonic lethality, and sterility. Data for Halo::PAR-3 fusions shown (see Fig. S1 for mNG::PAR-3). **(C)** Daughter cell asymmetry (Area^AB^/Area^AB + P1^). **(D)** Percentage of embryos reaching L4 stage. **(E)** Surviving adults exhibiting sterility. Individual embryos (C) or replicates (D and E) shown with mean indicated. **(F)** PAR-3(∆69–82) exhibits increased diffusivity relative to PAR-3(WT). HaloTag labeling was used to sparsely label PAR-3(∆69–82) and effective diffusivity calculated from mean perpendicular step size for τ = 0.2 s (WT: 12,810 particles, 13 embryos, ∆69–82: 3,616 particles, 14 embryos). (i and ii) Distribution of diffusivities for all molecules, mean indicated (i) and mean diffusivity per embryo, means indicated (ii) (WT data reproduced from Fig. 2 G for comparison). **(G)** PAR-3(∆69–82) displacements are biased along the flow axis. Plots of per-embryo mean displacements parallel and perpendicular to the flow axis for τ = 0.5 s. **(H)** PAR-3(∆69–82) couples to cortical flow. Coupling coefficients for PAR-3(∆69–82) shown for all molecules or molecules binned by diffusivity shown compared to PAR-3(WT) and NMY-2. Datapoints for PAR-3(WT) and NMY-2 represent single embryos. Due to reduced numbers of molecules per embryo for PAR-3(∆69–82) embryos, each data point in PAR-3(∆69–82) bins reflects molecules combined from multiple embryos to obtain sufficient numbers for fitting. Due to small sample size, significance assessed by Mann–Whitney U test.

**Figure S1.**
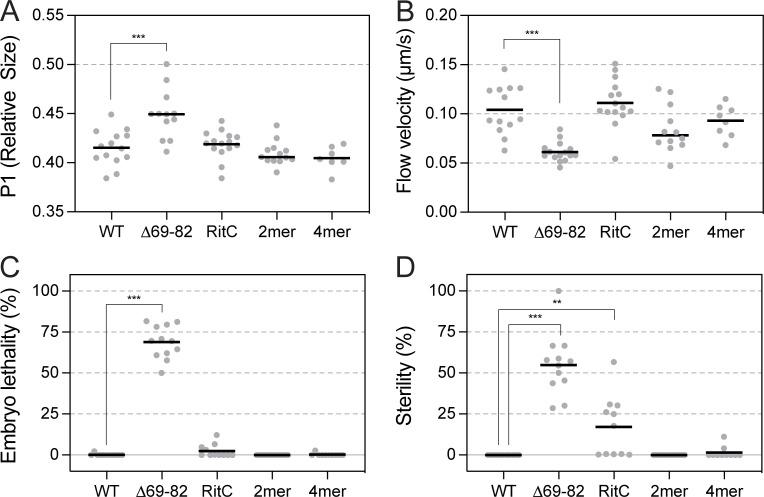
**Rescue of PAR-3(∆69–82) phenotypes by RitC, 2mer, and 4mer domains. (A)** P0 division size asymmetry expressed as relative size of P1 as calculated from area of P1 and AB in a midsection image taken after cytokinesis (Area^P1^/Area^P1+AB^). **(B)** Cortical flow velocity as measured by NMY-2 (PIV analysis). **(C and D)** Embryonic lethality (C) and sterility (D) of F1 progeny of animals homozygous for the indicated *par-3* allele. Significance vs. WT analyzed with multiple comparison correction (A and B, ANOVA with Dunnett’s correction; C and D, Kruskal–Wallis with Dunn’s correction), with only P < 0.05 shown. ***P < 0.001, **P < 0.01.

Finally, it remained possible that even the smallest particles that we could detect at the membrane were oligomers of some form and that formation of a minimal oligomer size is nonetheless required for advection, which would be consistent with the observations of Chang and Dickinson (2022). Oligomerization depends on the conserved CR1 oligomerization domain (Benton and St. Johnston, 2003; Feng et al., 2007; Mizuno et al., 2003; Zhang et al., 2013), mutation of which disrupts PAR-3 localization and leads to polarity defects and embryonic lethality (Dickinson et al., 2017; Li et al., 2010). We therefore introduced a small, previously characterized deletion into the endogenous *par-3* locus to generate an oligomerization defective PAR-3, PAR-3(∆69–82) (Fig. 3 A). Similar to prior results using this or similar mutations, introduction of the ∆69–82 mutation resulted in failure of PAR-3 to segregate into the anterior membrane and an overall loss of membrane association, as well as high rates of symmetric P0 divisions, embryonic and adult lethality, and sterility (Fig. 3, B–E). Consistent with the role of aPARs in stimulating contractility, *par-3*(∆*69–89*) embryos also exhibited partially reduced cortical flow (Fig. S1 B; Dickinson et al., 2017; Li et al., 2010; Munro et al., 2004; Rodriguez et al., 2017).

While membrane association was reduced to the point of being nearly undetectable over cytoplasmic background when using a mNG::PAR-3(∆69–82) fusion, single molecule analysis using labeling of a Halo-tagged allele allowed us to track sufficient numbers of membrane binding events for analysis (Video 1). Overall, PAR-3(∆69–82) molecules exhibited faster mean diffusivity compared to wild-type PAR-3, consistent with reduced clustering (Fig. 3 F). Strikingly, the coupling coefficient for monomeric PAR-3 was only modestly reduced compared to wild type (>0.7 vs. 0.85; Fig. 3, G and H). The fact that coupling coefficients remained high and consistent over all diffusive bins analyzed indicate that the failure of PAR-3(∆69–82) to exhibit significant segregation is not due to an inability to couple to cortical flows.

**Video 1.** **Monomeric PAR-3 at the cortex undergoes advection.** Near-TIRF timelapse image sequence of Halo::PAR-3(Δ69–82). Playback, 200×, scale bar = 10 µm).

### Rescue of cluster-defective PAR-3

**Figure 4.**
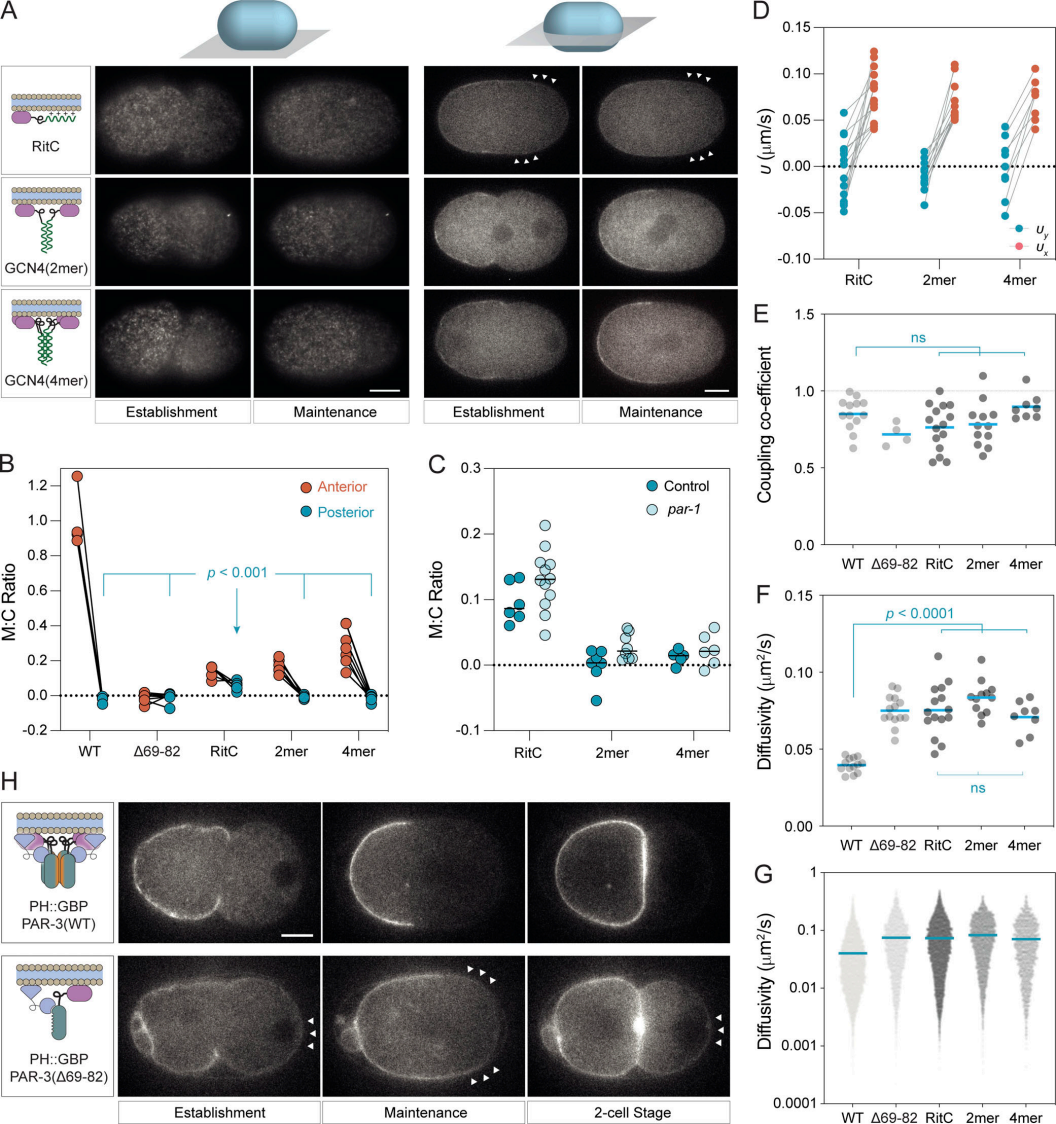
**Rescue of monomeric PAR-3 segregation by ectopic membrane targeting and/or clustering. (A)** Schematic of rescue constructs fused to mNG::PAR-3(∆69–82) shown along with example images of embryos taken at the cortex or at midplane at establishment and maintenance phases. Arrowheads indicate regions with posterior localization (RitC). Note midplane images are shown with identical intensity scaling to highlight relative levels, while cortex images have been individually scaled to improve visibility of the clusters at the cortex. Scale bars = 10 µm. **(B)** Quantification of membrane to cytoplasm (M:C) ratio at the anterior and posterior pole at maintenance phase highlights reduced ability of PAR-3(RitC) to remain excluded from the posterior pole compared to clustered variants (Tukey’s multiple comparison test). **(C)** Posterior clearance is unaffected by *par-1*(*RNAi*). M:C ratios at the posterior pole were calculated from embryos subject to *ctl* or *par-1*(*RNAi*). Mean values indicated. Differences between *ctl* and *par-1* were not significant, unpaired *t* test, Holm-Šídák correction. **(D)** Fit values for advection velocity parallel (*υ*_*x*_, red) and perpendicular (*υ*_*y*_, blue) relative to the flow axis are similar for all rescued variants. Lines connect paired values from single embryos. **(E)** Coupling coefficients are similar for all variants. Coupling coefficients calculated for all molecules in individual embryos are shown with means. Tukey’s multiple comparison test—variants are not significantly different from WT or between themselves. **(F and G)** All PAR-3 variants exhibit similar increases in diffusivity relative to PAR-3(WT). HaloTag labeling was used to sparsely label PAR-3 variants and effective diffusivity calculated from mean perpendicular step size for τ = 0.2 s. Mean diffusivity per embryo (F) and for all molecules (G) shown with mean indicated (RitC: 11,692 particles, 15 embryos, 2mer: 5,519 particles, 12 embryos, 4mer: 2,376 particles, 8 embryos). WT and PAR-3(∆69–82) data reproduced from Fig. 3 F for comparison. Mean diffusivity per embryo values for variants are significantly different from wild type, but not from each other. Tukey’s multiple comparison test. **(H)** Effect of clustering on membrane-tethered PAR-3. GFP fusions to either PAR-3(WT) or PAR-3(∆69–82) were tethered to the membrane via a membrane-associated nanobody (PH::GBP) and their ability to segregate in response to flows tested. Note that PAR-3(WT) segregates normally and is fully excluded from the posterior pole despite constitutive targeting to the plasma membrane and is absent from the posterior P1 daughter cell. By contrast, PAR-3(∆69–82) is less efficiently cleared and partially reinvades the posterior once flows cease such that it is also present on the plasma membrane of the P1 daughter cell. Arrowheads highlight posterior GFP signal. Scale bar = 10 µm.

We next asked whether we could rescue the segregation of monomeric PAR-3(∆69–82) through replacing CR1-dependent oligomerization either with dimeric (2mer) or tetrameric (4mer) forms of the GCN4 leucine zipper to restore clustering or with an ectopic membrane targeting signal (RitC) to restore membrane localization independently of clustering (Fig. 4 A; Harbury et al., 1993; Onken et al., 2006; Matsuda et al., 2005).

We found that simply stabilizing membrane binding via RitC was sufficient to allow PAR-3 asymmetry to develop (Fig. 4 A, top). Segregation was noticeably less efficient than for PAR-3(WT) as levels on the posterior membrane remained significantly above zero (Fig. 4 B). Nonetheless, PAR-3(RitC) was able to rescue division size asymmetry, cortical flow velocity, and embryonic lethality observed in *par-3*(*∆69*–*82*) embryos, though only partially rescued adult sterility (Fig. S1). Notably, we observed strong coupling to cortical flows with a coupling coefficient that was not significantly different from wild type (Fig. 4, D and E; and Video 2). These data suggest that being monomeric per se does not impact coupling to the cortex and that the modestly reduced coupling of PAR-3(∆69–82) that we observed may be an artifact of reduced cortical flow velocities that are associated with defective PAR-3 function.

**Video 2.** **Comparison of advection of Halo::PAR-3 variants.** Near-TIRF timelapse image sequence of Halo::PAR-3(WT) compared to RitC, 2mer, and 4mer variants. Playback, 200×, scale bar = 10 µm).

As expected, PAR-3(2mer) and PAR-3(4mer) also showed wild-type levels of coupling to the cortex (Fig. 4, D and E). However, in contrast to PAR-2(RitC), both exhibited highly efficient segregation, with effectively no detectable signal remaining in the posterior half of the embryo, similar to PAR-2(WT) (Fig. 4, A and B). PAR-3(4mer) appeared noticeably more punctate and exhibited higher membrane concentrations compared to PAR-3(2mer) consistent with a larger oligomer size and increased membrane affinity. At the same time, both cluster size and membrane concentrations were substantially below that of PAR-3(WT), consistent with the ability of the CR1 domain to drive formation of large oligomers (compare Fig. 4 A vs. Fig. 3 B). Division asymmetry, cortical flow rates, embryonic lethality, and adult sterility were normal in both 2mer and 4mer variants (Fig. S1). Thus, ectopic oligomerization of monomeric PAR-3 mutants appears sufficient to rescue PAR-3 function and enhances segregation relative to membrane targeting alone.

We wondered whether this difference in behavior between PAR-3(RitC) and oligomeric forms of PAR-3 (i.e., WT, 2mer, 4mer) could be due to differential sensitivity to posterior membrane exclusion by the posterior PAR kinase PAR-1 as RitC introduces a PAR-1-insensitive membrane binding site. We therefore repeated our analysis in embryos depleted for PAR-1 by RNAi. We found that membrane concentrations were slightly elevated in *par-1(RNAi)* in all cases, but the relative efficiency of segregation of the different variants was unchanged, indicating that PAR-1 activity is not a key determinant of segregation efficiency when flows are present (Fig. 4 C).

Finally, to confirm that ectopic membrane targeting per se does not compromise PAR-3 segregation behavior, we induced ectopic membrane targeting of GFP fusions to wild-type and monomeric PAR-3 by co-expressing a membrane-associated anti-GFP nanobody (PH::GBP; Rodriguez et al., 2017). Membrane-tethered monomeric PAR-3(∆69–82) behaved similarly to PAR-3(RitC), exhibiting modest segregation. By contrast, membrane-tethered PAR-3(WT) was segregated as efficiently as wild type (Fig. 4 H).

On the basis of these experiments, we conclude that membrane stabilization appears largely sufficient to rescue PAR-3(∆69–82) function and enable PAR-3 segregation and division asymmetry. Why oligomerization yields improved segregation over membrane-targeted monomers is unclear but does not appear to be related either to differential abilities of these molecules to couple to motion of the cortex or to oligomer-dependent changes in diffusivity: All rescued variants of PAR-3 examined were advected by flows with similar velocities, and segregation efficiency was not correlated with differences in diffusivity (Fig. 4, F and G). Given that all oligomeric forms of PAR-3 exhibited higher membrane concentrations compared to PAR-3(RitC), it seems most likely that segregation efficiency relates to oligomerization-dependent changes in membrane binding kinetics.

### Differential coupling of anterior and posterior PAR proteins to cortical flow does not explain differences in segregation

While differential coupling to the cortex could not explain the dependence of PAR-3 segregation on its oligomeric state, it remained possible that PAR-3 itself is unique among PAR proteins in its ability to couple to cortical flows. Such a model could explain why segregation of PAR-6 and PKC-3 is dependent on their ability to associate with PAR-3 clusters (Rodriguez et al., 2017), and similarly, why posterior PAR proteins are generally not strongly segregated into the anterior, even under conditions in which they are constitutively membrane-associated during the period of cortical flow (Folkmann and Seydoux, 2019; Hao et al., 2006; Rodriguez et al., 2017).

**Figure 5.**
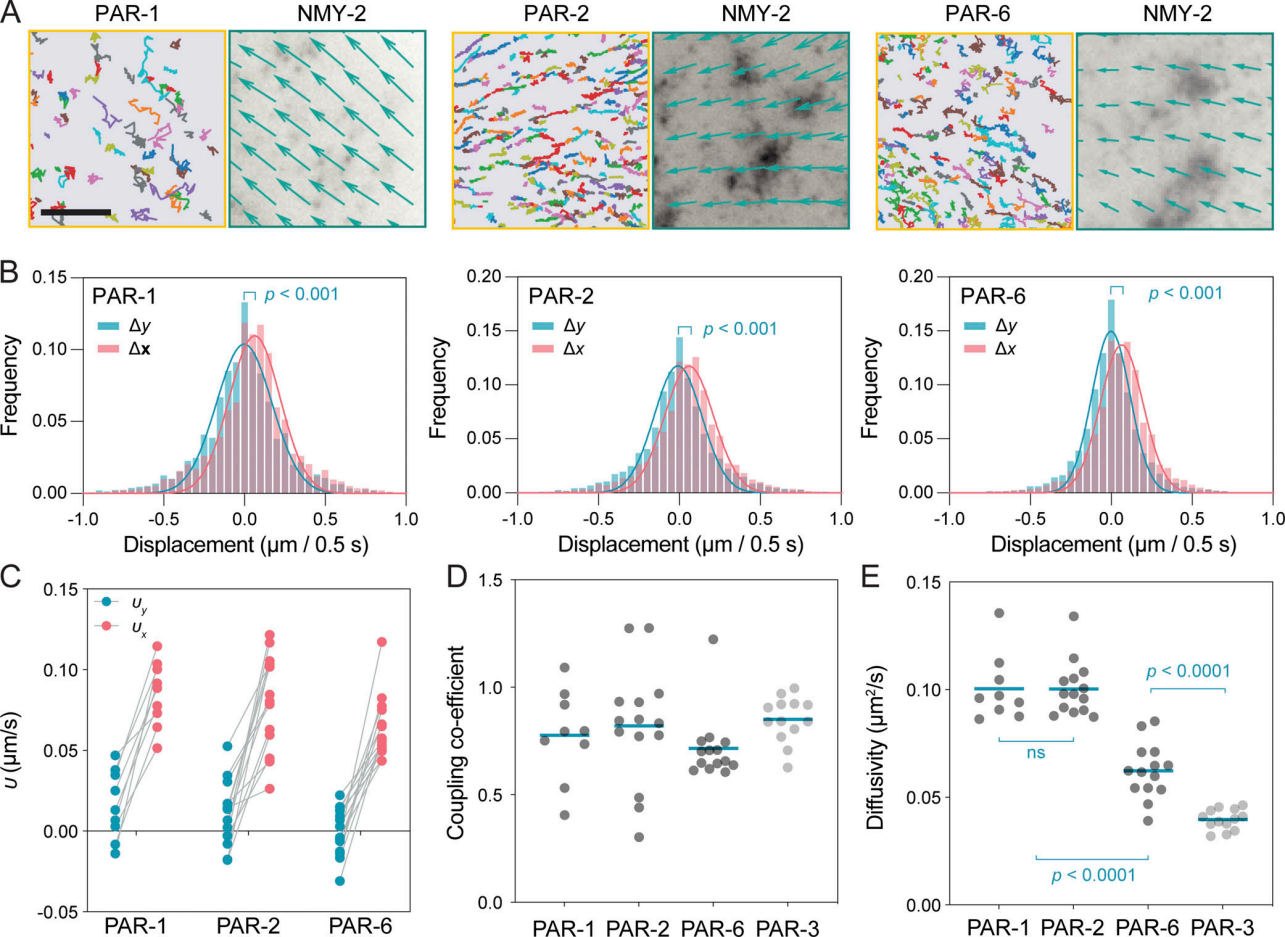
**Advection is a general property of PAR proteins. (A)** Sample trajectories (>15 frames) from a region in the embryo posterior shown for HaloTag-labeled PAR-1, PAR-2, and PAR-6. Corresponding flow fields derived from PIV analysis of NMY-2 shown on the right. Vector lengths were normalized to highlight direction of vectors. Scale bar = 5 µm. **(B)** Plots of displacements parallel and perpendicular to the flow axis fit with a Gaussian distribution. Difference calculated via unpaired *t*-test, two-tailed. **(C)** Fit values for advection velocity for PAR-1, PAR-2, and PAR-6 from displacements parallel (*υ*_*x*_, red) and perpendicular (*υ*_y_, blue) to the local flow axis highlight drift along the flow axis (τ = 0.5 s). Lines connect paired data from single embryos. **(D)** Coupling coefficients for PAR-1, PAR-2, and PAR-6 are similar to those observed for PAR-3. Individual embryo data points and mean shown. **(E)** Mean diffusivity per embryo (τ = 0.2 s) for PAR-1, PAR-2, PAR-3, and PAR-6 highlight lack of correlation between diffusivity and coupling coefficient. Note, PAR-3 data reproduced from Fig. 3 F for comparison. Tukey’s multiple comparison test.

To determine whether other PAR proteins exhibit coupling to cortical flows, we applied our analysis to sparsely labeled Halo-tagged fusions to PAR-1, PAR-2, and PAR-6 during the polarity establishment phase. In all three cases, molecules could clearly be seen moving toward the anterior during the period of cortical flow (Fig. 5 A and Video 3). Plots of displacements revealed a clear drift parallel to the local flow vector, consistent with all three proteins being advected by cortical flow over short timescales (Fig. 5, B and C). For all three proteins, coupling coefficients were similar and generally exceeded 0.7 (Fig. 5 D). Both PAR-1 and PAR-2 exhibited higher mean diffusivity compared to PAR-6, which was itself significantly higher than that observed for PAR-3, again consistent with a general lack of correlation between diffusivity and the ability of molecules to couple to cortical flows (Fig. 5 E). Thus, selective coupling to the cortex cannot account for the differential ability of PAR proteins to be segregated by cortical flows.

**Video 3.** **Advection is a general property of PAR proteins.** Near-TIRF timelapse image sequence of Halo fusions to PAR-1, PAR-2, and PAR-6. Playback, 200×, scale bar = 10 µm).

### A simple model for advective transport predicts segregation efficiency

To gain insight into the key determinants of segregation efficiency, we considered a simple model in which molecules undergo diffusion, membrane association/dissociation from a uniform cytoplasmic pool, and advection. Given the lack of effect of oligomer size on relative advection velocity, the simplest explanation to explain differential segregation would be through effects of oligomerization on the diffusivity (*D*) and/or membrane dissociation rate (*k*_off_). Generally, oligomerization should result in both a larger molecule, hence reduced diffusivity, and reduced membrane dissociation due to increased membrane avidity, both of which would generally be expected to enhance segregation.

Taking account of our results so far, we imposed a spatially varying velocity function that was fit to experimentally determined flow fields and varied *D* and *k*_off_ across multiple orders of magnitude. To isolate the effects of *D* and *k*_off_, we did not include any feedback which could amplify or destabilize asymmetries. We applied an advection regime in which flow was active for a period of 500 s, corresponding to the 5–10 min polarity establishment phase during which flows initially pattern the PAR proteins (Blanchoud et al., 2015; Goehring et al., 2011b; Gross et al., 2019). Subsequently, to examine the persistence of asymmetry during the period after flows cease and before cytokinesis during which polarity is maintained, we then set the velocity function to zero at all positions and allowed the system to equilibrate. We scored both segregation efficiency, quantified by the peak magnitude of asymmetry (ASI, asymmetry index; higher ASI = more asymmetry) and the relative posterior depletion after the 500 s period of cortical flow, as well as the persistence of asymmetry as measured by the timescale of asymmetry decay during the flow-independent maintenance phase.

**Figure 6.**
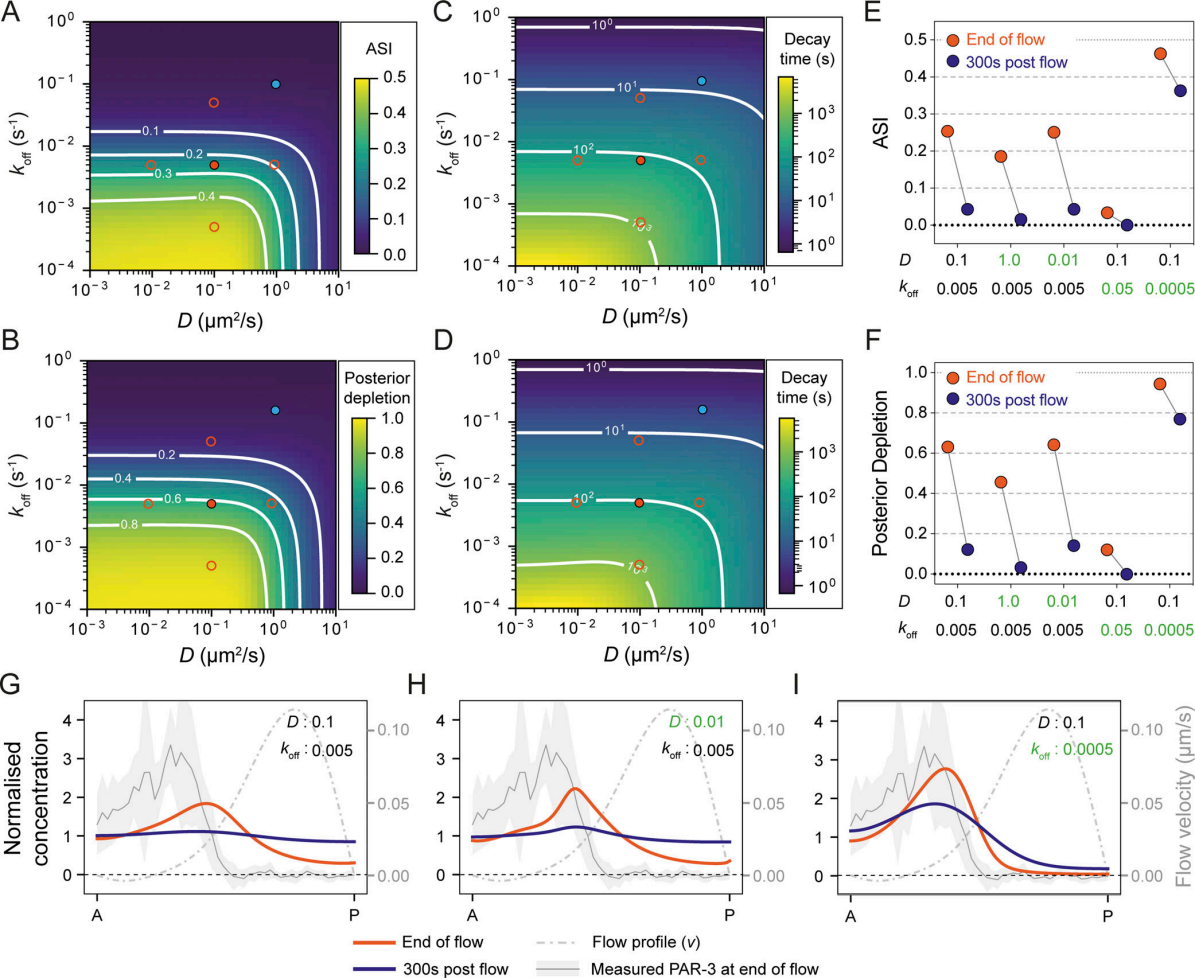
**Predicted efficiency of advective segregation as a function of *D* and *k***_**off**_**. (A–D)** The efficiency of advective segregation of a hypothetical molecule as a function of *k*_off_ and *D* when subjected to an experimentally fit flow profile for a period of 500 s. **(A and B)** show maximum ASI and relative posterior depletion at the end of 500 s of flow, respectively. **(C and D)** show the characteristic time (*t*_1/2_) for decay in ASI and posterior depletion after flow ceases. Red circles in A–D indicate example parameter sets approximating the measured behavior of PAR-2 (solid circle, *D* ∼ 0.1 µm^2^/s, *k*_off_ ∼ 0.005 s^−1^) along with 10-fold increases or decreases in *D* and *k*_off_ (open circles). Blue circle indicates expected behavior for the PH domain of PLC1δ1, which binds PIP_2_ and which we previously have shown does not segregate in embryos (*D* ∼ 1.0 µm^2^/s, *k*_off_ ∼ 0.1 s^−1^; Hirani et al., 2019). **(E and F)** ASI (E) and posterior depletion (F) shown at the end of flow and 300 s later, equivalent to the end of establishment phase and post-NEBD-maintenance phase, respectively, for the example parameter sets denoted in A–D. Note that the magnitude and stability of asymmetry are strongly impacted by 10-fold changes in *k*_off_, but not similar changes in *D*. **(G–I)** Example concentration profiles at the end of flow and 300 s post flow for the indicated parameter sets from A–F. The flow velocity function and measured PAR-3 distribution (mean ± SD) at the end of flow are shown for reference. Note that the parameter varied in H and I vs. G is indicated in green, highlighting how reductions in *k*_off_, but not *D*, improve segregation.

As expected, segregation efficiency, measured either by ASI or posterior depletion, was maximized as *k*_off_ and *D* were reduced (Fig. 6, A and B), as was the stability of the induced asymmetry (Fig. 6, C and D). Thus, one would expect little to no segregation for relatively rapidly diffusing and exchanging molecules. Indeed, as we have previously shown, a small lipid (PIP_2_) binding probe PH(PLCδ1) showed no segregation in response to cortical flow, consistent with the predictions here (Fig. 6, A–D; Hirani et al., 2019).

PAR proteins typically exhibit diffusivities characterized by *D* ≤ ∼0.1 µm^2^/s. In this regime, further reductions in *D* made little difference and consequently, cluster-induced reductions in diffusivity of PAR-3 (e.g., from *D* ∼ 0.08 to *D* < 0.005 µm^2^/s) would be expected to have only very minor effects on segregation efficiency (<2%; Fig. 6, E–H). This result is consistent with diffusivity being a poor predictor of differential segregation of our PAR-3 variants (see Fig. 4).

By contrast, in this regime, segregation was highly sensitive to *k*_off_. Notably, segregation was effectively non-existent for *k*_off_ > 0.1 s^−1^, while segregation was highly efficient for *k*_off_ < 0.001 s^−1^. Similarly, the rate of asymmetry decay decreased with reductions in *k*_off_ (Fig. 6, E–G and I). Thus, in the absence of additional feedback and for typical diffusivities of PAR proteins, both the magnitude of asymmetry in response to cortical flows and its persistence once flows cease is predicted to be primarily set by how long molecules remain associated with the membrane.

**Figure S2.**
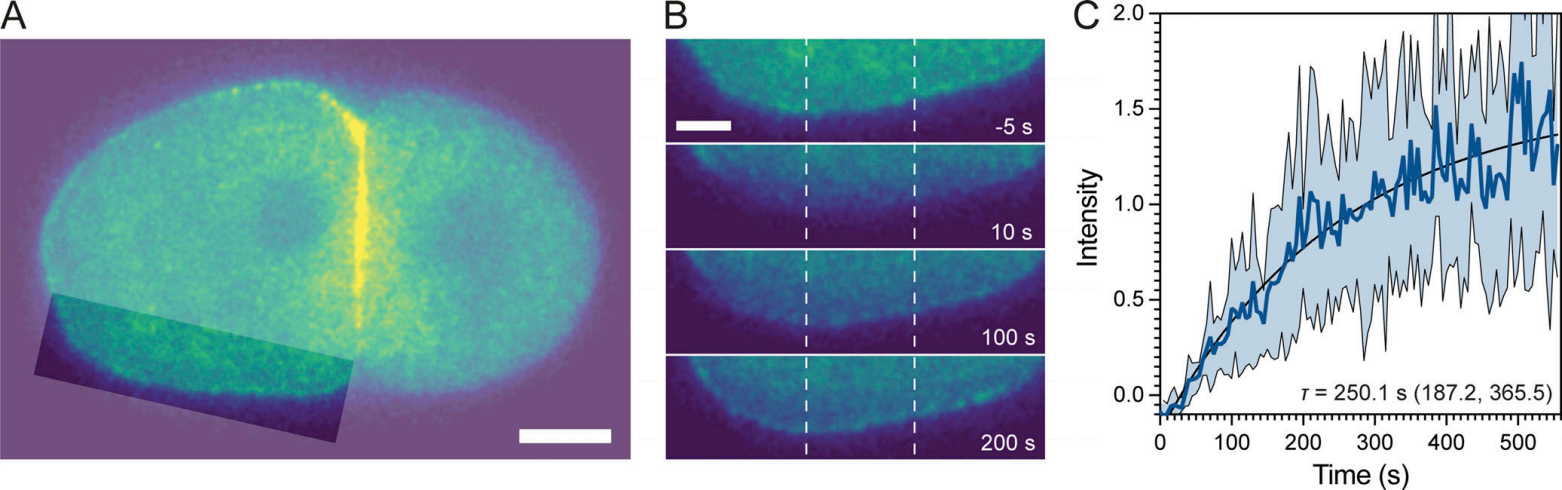
**FRAP analysis of bulk PAR-3 turnover on the membrane.** Two-cell embryos expressing mNG::PAR-3 were subject to targeted photobleaching (∼0.4 s) along one edge of the anterior AB cell (highlighted regions, A). **(A and B)** Images show the entire embryo before bleaching (A, scale bar = 10 µm) and insets highlighting the bleached zone for the indicated times relative to photobleaching (B, scale bar = 5 µm). **(C)** Quantification of membrane fluorescence within the center of the bleached region (B, dashed lines) shown relative to a corresponding unbleached control region and normalized to pre-bleach intensity. Mean ± SD shown (*n* = 6). Data fit to a one-component association model to obtain the characteristic turnover time, τ (1/*k*_off_), shown with 95% CI.

Our and others’ prior measures of bulk PAR protein turnover at the membrane have yielded values for *k*_off_ of <0.01 s^−1^, equating to characteristic turnover timescales of >100 s (Goehring et al., 2011a; Hubatsch et al., 2019; Robin et al., 2014). While analysis of bulk PAR-3 turnover is complicated by cell-cycle-dependent changes in membrane binding (Dickinson et al., 2017; Rodriguez et al., 2017), our preliminary analysis indicates that it occurs on a similar timescale (Fig. S2). Thus, the kinetic parameters governing the behavior of PAR proteins at the membrane appear to be tuned to a region in parameter space that ensures both that cortical flows will have the capacity to significantly impact their distributions in cells, and that the effects of this advective transport can be modulated by changes in membrane binding dynamics.

A key prediction of this model therefore is that long-range transport by cortical flows is not a unique feature of PAR-3 but should also be observed for other PAR proteins. Indeed, for the measured membrane dynamics of PAR-2 and PAR-6 (*D* ∼ 0.1 µm^2^/s, *k*_off_ ∼ 0.005 s^−1^), the model predicts modest and transient of segregation (ASI ∼ 0.25, posterior depletion ∼ 0.6, *t*_1/2_ ∼ 100 s; Fig. 6, A–G; parameters from this work and Goehring et al., 2011a; Hubatsch et al., 2019; Robin et al., 2014).

**Figure S3.**
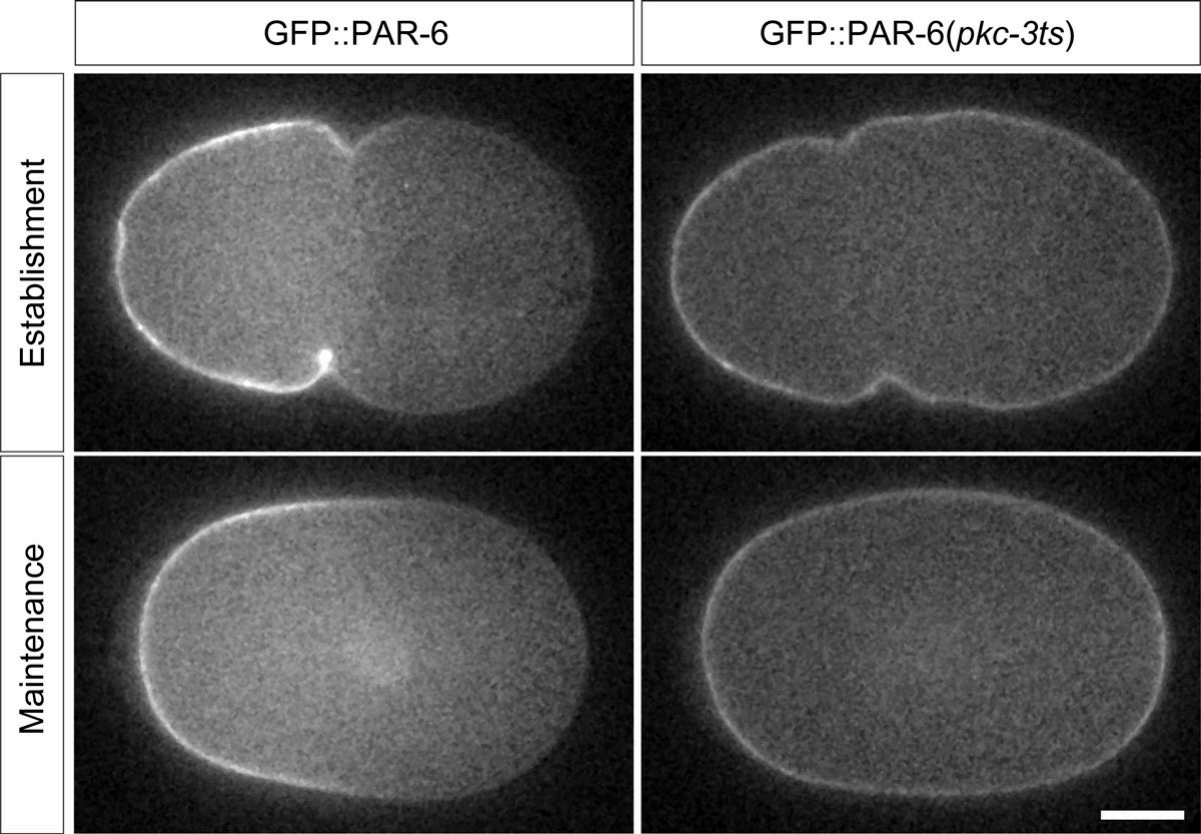
**PAR-6 undergoes weak and transient asymmetry when decoupled from PAR-3 clusters.** Representative midplane confocal images of PAR-6::GFP in wild-type or a *pkc-3(ts)* background in which PAR-6 membrane association is decoupled from PAR-3. When PAR-6 is not associated with PAR-3 clusters, segregation still occurs but is weaker and more transient.

Because PAR-6 associates with PAR-3 clusters and PAR-3 is normally required for both PAR-6 membrane association and cortical flows, we could not measure PAR-3-independent segregation of PAR-6 in a wild-type context. However, inhibition of PKC-3 activity via a temperature-sensitive allele allows PAR-6 and PKC-3 to associate with the membrane in a PAR-3–independent manner, through a yet undetermined mechanism (Rodriguez et al., 2017). We find that in these conditions PAR-6 is still able to acquire some asymmetry, though to a substantially lower degree compared to when it associates with PAR-3 clusters. Further, unlike PAR-3, the resulting asymmetry decayed once flows ceased, consistent with model predictions (Fig. S3).

**Figure 7.**
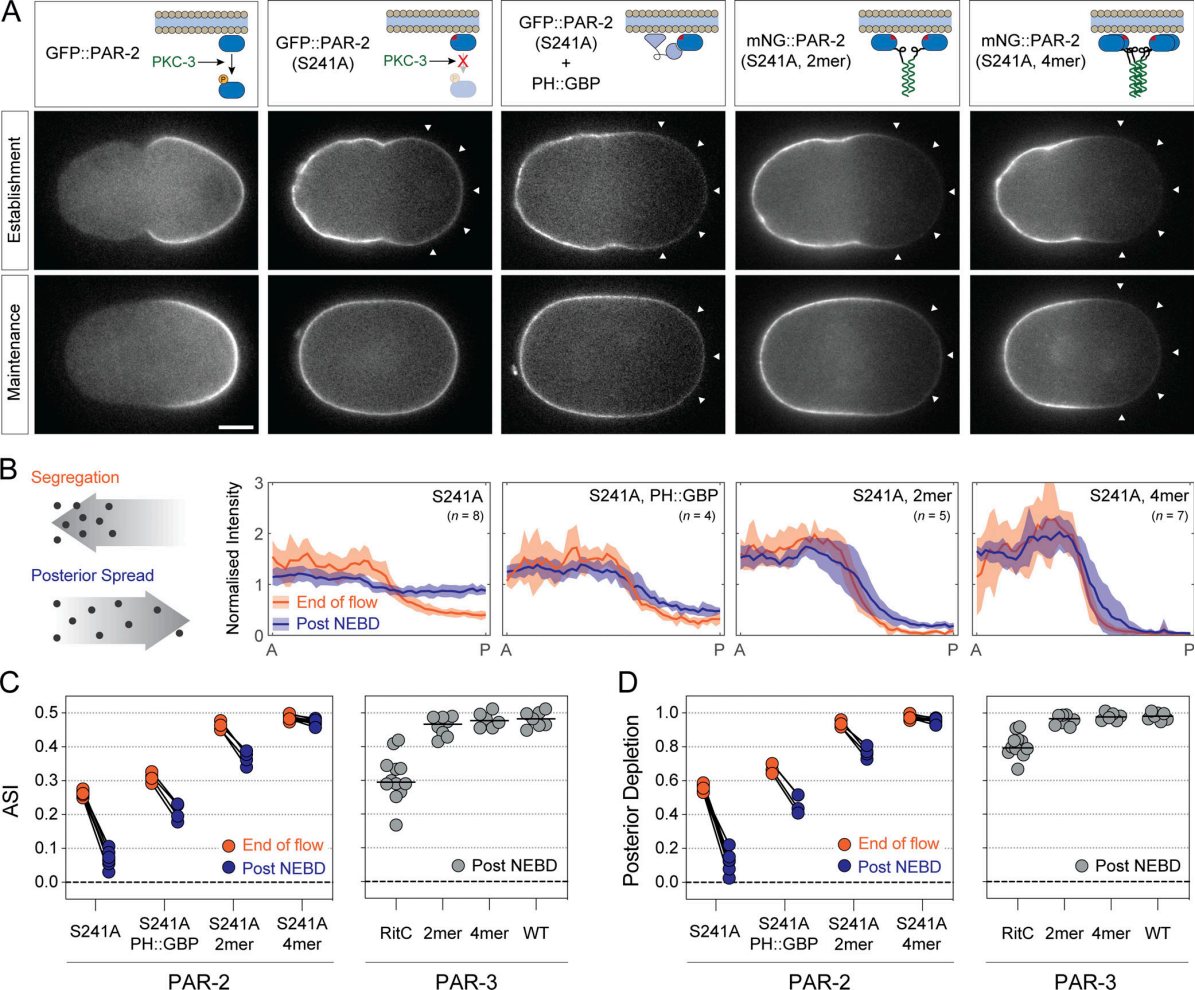
**Reducing membrane turnover anteriorizes posterior protein PAR-2. (A)** Mutation of the PKC-3 target site in PAR-2, PAR-2(S241A), leads to modest transient anterior enrichment of PAR-2. Note reversed asymmetry compared to PAR-2(WT). The magnitude and stability of anterior segregation is enhanced by tethering to the membrane (PH::GFP) or by oligomerization (2mer, 4mer). Representative midplane confocal images captured at the end of the establishment phase (End of flow) and maintenance phase (Post NEBD) highlighting peak segregation and degree of asymmetry decay once flows cease. **(B)** Quantification of membrane profiles of zygotes expressing PAR-2(S241A) variants shown in A. Mean ± SD shown. **(C and D)** Quantification of ASI and relative posterior depletion for PAR-2(S241A) variants plotted along with Post NEBD data for PAR-3 zygotes for comparison (see Fig. 4). Note that both oligomeric forms of PAR-2(S241A, 2mer/4mer) achieve near maximal levels for both ASI (0.5) and posterior depletion (1.0) during the establishment phase, which decay only minimally once flows cease. Data for S241A are pooled from GFP:: and mNG::PAR-2(S241A), which behaved identically.

Similar to PAR-6, we also could not analyze PAR-2 segregation in wild-type conditions as it is actively excluded from the anterior by PKC-3. Thus, we introduced an alanine mutation into the key PKC-3 phosphosite, S241, which has been shown to prevent PKC-3-dependent displacement of PAR-2 from the plasma membrane and was previously reported to lead to uniform PAR-2 localization to the membrane (Hao et al., 2006; Motegi et al., 2011). We found that PAR-2(S241A) was in fact transiently enriched in the anterior by cortical flows, reaching a peak ASI of ∼0.26 and peak depletion of PAR-2(S241A) in the posterior of ∼0.56, figures that quantitatively matched the predictions of our model (Fig. 7 A, Establishment; 7, B–D, End of Flow). This asymmetry then dissipated substantially after flows ceased. Both ASI and peak depletion decayed by >70% within ∼300 s, again consistent with predictions of our model (Fig. 7 A, Maintenance; B). Thus, the segregation behavior of PAR-2 and PAR-6 is fully consistent with predictions of this simple model of advective segregation.

**Figure S4.**
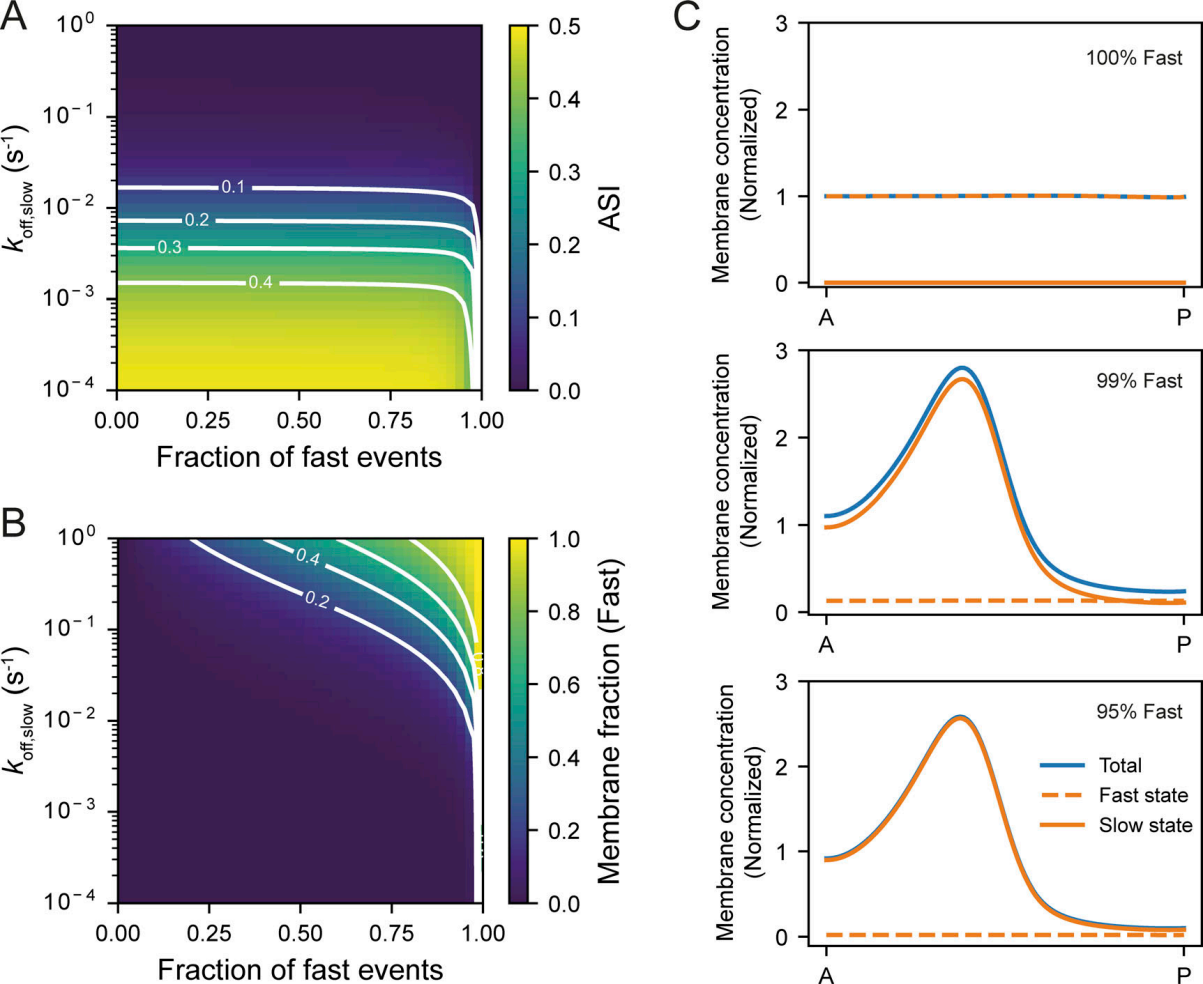
**Slow-exchanging molecules can dominate the membrane-associated pool of molecules and define the response of the system to cortical flows even if constituting a minority of membrane binding events.** To assess the response of a system with a mixture of fast- and slow-exchanging molecules to advective transport, we modified the model in Fig. 6 to allow molecules that bind to the membrane to exhibit a mix of membrane-bound lifetimes: short (fast-exchanging, *k*_off, fast_) and long (slow-exchanging, *k*_off,slow_). Fast events were fixed at *k*_off, fast_ = 1 s^−1^ and *k*_off,slow_ varied. **(A and B)** The relative membrane fraction constituted by fast-exchanging molecules (A) and the overall asymmetry (ASI) achieved at the end of flow (B) are shown as a function of the relative fraction of fast membrane binding events and the relative values of *k*_off, fast_ and *k*_off,slow_. **(C)** Example traces showing the distribution of fast, slow, and total molecules shown for the indicated fraction of fast events for *k*_off,slow_ = 10^−3^ s^−1^. To briefly summarize, in C, the 1,000-fold-longer persistence of slow-exchanging molecules at the cortex means that they make up the vast majority of molecules at the membrane at any given time even when they constitute only 1% of all membrane binding events. Consequently, the overall asymmetry achieved by advection is dominated by the response of the long-lived species.

We would note that others have reported substantially reduced lifetimes of membrane association for both PAR-2 and PAR-3. These measurements were derived from single-molecule tracking and implied mean lifetimes ∼1 s, which generally would be incompatible with long-range transport by cortical flows (Arata et al., 2016; Chang and Dickinson, 2022). While it is possible that single particle tracking methods fail to capture true off rates due to limits imposed by photobleaching and tracking error, these divergent measurements may be reconciled if molecules exhibit a mix of fast- and slow-exchanging behaviors as particle- and ensemble-based methods of measuring turnover will differentially weight these pools. Indeed, both prior studies reported sub-populations of molecules with membrane dwell times substantially longer than the mean behavior, consistent with the existence of long-lived species. To examine the effects of advection on a system comprising a mixture of molecules with different membrane-bound lifetimes, we repeated our simulations but assigned molecules at the membrane stochastically into either fast- or slow-exchanging states. We found that due to the effects of time weighting, slow-exchanging molecules can easily dominate the pool of membrane-associated molecules, even when fast-exchanging molecules constitute the majority of membrane-binding events (Fig. S4). Thus, we would argue that the slow exchanging pool reported on by bulk turnover measurements is likely to dictate the response of the system to cortical flows.

### Tuning membrane turnover is sufficient to “anteriorize” posterior PAR protein PAR-2

If stability of membrane binding is the key determinant of efficient PAR segregation, as we suggested, one should be able to tune segregation efficiency by varying membrane affinity. To explicitly test this prediction, we stabilized membrane association of PAR-2(S241A) either by tethering it to the membrane via co-expression of a GFP::PAR-2(S241A) fusion with a membrane targeted GFP binding protein (PH::GBP) or by oligomerization using 2mer or 4mer forms of the GCN4 leucine zipper, which effectively increases the number of membrane binding domains as a function of oligomer size (Fig. 7 A, top).

**Figure S5.**
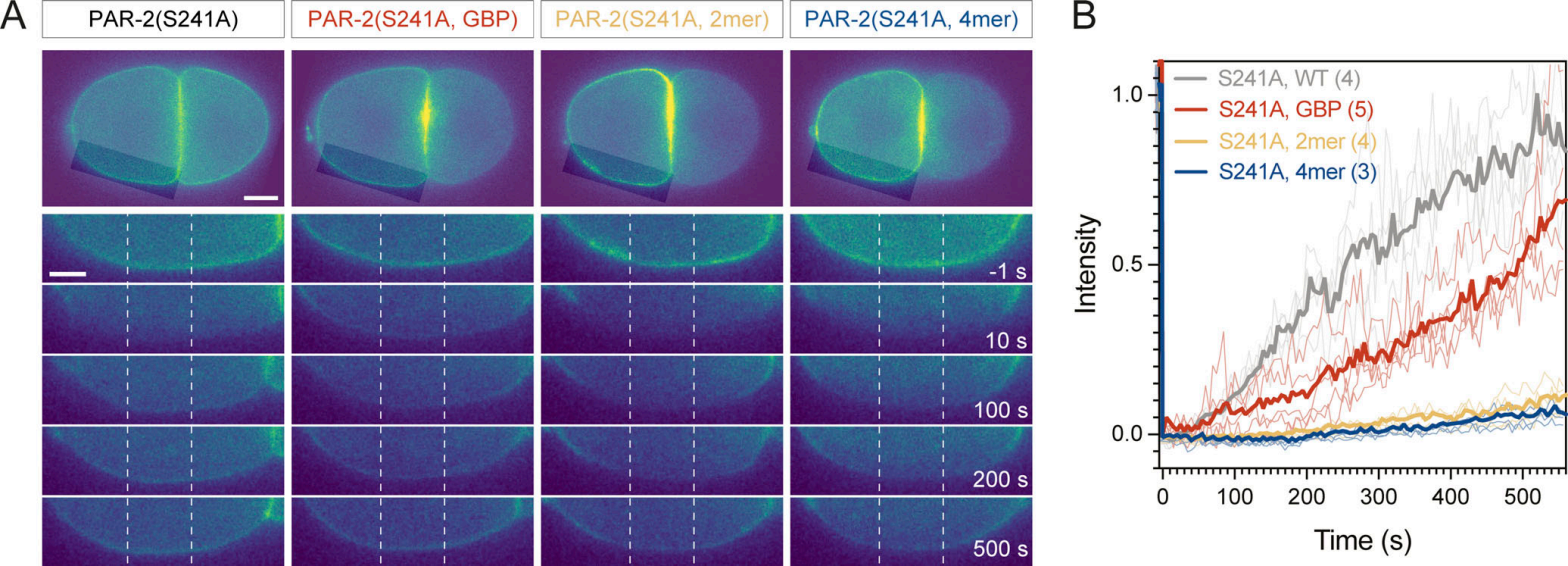
**Constitutive oligomerization of PAR-2 reduces membrane turnover. (A)** Two-cell embryos expressing the indicated mNG::PAR-2(S241A) alleles or membrane tethered GFP::PAR-2(S241A)+PH::GBP were subject to targeted photobleaching (∼0.4 s) along one edge of the anterior AB cell (highlighted regions, A). Images show the entire embryo before bleaching (A, top, scale bar = 10 µm) and insets highlighting the bleached zone for the indicated times relative to photobleaching (A, bottom, scale bar = 5 µm). **(B)** Quantification of membrane fluorescence within the center of the bleached region (A, dashed lines) shown relative to a corresponding unbleached control region and normalized to pre-bleach intensity. Mean shown along with individual replicates (*n* indicated). Note that reduced turnover correlates with better segregation (4mer > 2mer > GBP > WT, compare to segregation data in Fig. 7).

We found that either method of stabilizing membrane association resulted in clear increases in both peak asymmetry of PAR-2(S241A) as measured by ASI or posterior depletion at the end of the polarity establishment phase, as well as in the persistence of asymmetry once flows ceased in the polarity maintenance phase (Fig. 7, A–D). Photobleaching analysis confirmed that the magnitude of segregation generally increased with reductions in membrane turnover, with oligomeric forms of PAR-2(S241A) showing the most reduced turnover and most efficient segregation (Fig. S5). Notably, PAR-2(S241A, 4mer) achieved peak asymmetries of >0.46 and peak depletion in the posterior of >0.93, which were comparable to that observed for oligomeric forms of PAR-3 (ASI > 0.47, posterior depletion > 0.97; see Fig. 7, C and D, for comparison), with a profile at the end of flows being conspicuously similar to PAR-3 (compare Fig. 7 B, 241A, 4mer and Fig. 6 I, PAR-3). Thus, modulation of membrane binding appears sufficient to achieve efficient and persistent anterior segregation of PAR-2 by cortical flows, effectively reprogramming PAR-2 to achieve the pattern of polarization exhibited by oligomeric PAR-3.

## Discussion

While cortical flow-associated polarized transport has been observed for over a half century, the design features of molecules that determine whether molecules “go with the flow” have remained contentious. Experiments in non-adherent cells have proposed that cortical flows can induce bulk flow of plasma membrane components, including lipids, toward the cell rear leading to a treadmilling-like behavior. In such models, asymmetries arise from differential diffusion and/or internalization from the plasma membrane (Bretscher, 1996; O’Neill et al., 2018; Tanaka et al., 2017). However, in other systems, coupling to actin flow appears selective with no evidence for lipid flow, suggesting that molecules must differ in the degree to which their motion is entrained by the cortex (Lee et al., 1990; Sheetz et al., 1989). In both systems, clustering could be expected to tune segregation. In the former, the primary effects of clustering would be envisioned to impact diffusivity or membrane turnover, while in the latter, clustering could promote advection by altering the effective friction with the underlying cortex, for example through size-dependent entanglement with the cortical actin meshwork or other molecules linked to it.

Here, we have shown that PAR proteins are indeed subject to differential transport by cortical flows in a manner that correlates with clustering. However, differential transport does not arise through changes in advective coupling to the actin cortex. While we cannot exclude small differences in coupling efficiency, all molecules examined appeared to couple well to flows (*cc* > 0.7). Notably, there is no obvious correlation between the effective advective velocity of a molecule and either its oligomer size or diffusivity, which one might expect if mobility were defined by differential corralling by the cortical actin meshwork.

Indeed, the similar degree to which diverse PAR proteins couple to the cortex despite differences in diffusivity suggests that it is unlikely that corralling by the actin cortex is sufficient to determine whether a molecule is advected in this system. Whether this near-universal coupling of PAR proteins to cortical actin flows points toward a paradigm of coupled bulk membrane/cortex flow remains unclear (a nice discussion of membrane flows can be found in Jacobson and Kapustina, 2019). In support of such a model, none of the PAR proteins show obvious colocalization with actin and do not require actin to stabilize their localization to the membrane (Chang and Dickinson, 2022; Goehring et al., 2011a; Munro et al., 2004). However, lateral mobility of PAR-3 is enhanced by depolymerization of actin (Sailer et al., 2015) and we cannot rule out the possibility that PAR proteins may nonetheless be transiently linked to the actin cortex, perhaps through association into higher order, heterogenous assemblies with other proteins or lipids at the membrane:cortex interface. Resolving the nature of the coupling between the membrane and the underlying cortex and defining the molecular features that enable advection of membrane-associated molecules in this system will require a more comprehensive survey of the transport of different membrane-associated molecules.

Rather than driving differential coupling to the cortex, our data suggest that a key role of oligomerization is to facilitate membrane association, which in this system sets the timescales over which the effects of advection are integrated, and flow-induced asymmetries are retained after flows cease. In this sense, oligomerization enhances a molecule’s effective memory of cortical flows in a manner consistent with recent theoretical analysis of the role of oligomerization in polarity circuits (Lang and Munro, 2022). Importantly, we have shown that modulation of this memory allows one to invert the polarity of a normally posterior PAR protein.

Such a model of advection-driven segregation requires that advection timescales be sufficiently long to ensure transport across cellular length scales. As we show, for observed cortical flow velocities (∼0.1 µm/s) and embryo sizes (∼50 µm), the relevant particles (molecules or clusters) must exhibit lifetimes of membrane association in excess of ∼100 s, which is consistent with ensemble measurements of turnover times for PAR-2, PAR-3, and PAR-6 (this work and Goehring et al., 2010; Hubatsch et al., 2019; Robin et al., 2014), as well as the visible flow of PAR-3 clusters across the embryo over the course of minutes. While others have argued for much faster membrane turnover dynamics based on single particle analysis (Arata et al., 2016; Chang and Dickinson, 2022), our data indicate both that a slow-exchanging population must exist and that this slow-exchanging pool is likely to dictate the efficiency with which cortical flows can induce asymmetries in membrane concentration.

By contrast, our data suggest that oligomer-induced changes in diffusivity are not critical for differential segregation of PAR proteins in this system, presumably due to the large system size (50 µm) and the relative slow diffusive regime of PAR proteins (<0.1 µm^2^/s). For smaller system sizes and/or faster diffusing molecules, diffusion may limit effective segregation, in which case modulation of diffusivity by oligomerization could play a significant role. As we have shown, reductions in cell size can impact the ability of cells to polarize (Hubatsch et al., 2019). Moreover, in the cytoplasm, where mobilities are generally much higher, spatial modulation of diffusive mobility has been shown to be a key ingredient in the establishment of intracellular gradients (Daniels et al., 2009; Tenlen et al., 2008).

Finally, returning to the specific case of PAR-3 that motivated this work, it is important to note what our model can and cannot explain. Our data are generally consistent with a link between stabilizing membrane association and the ability of PAR-3 to segregate: first, stabilizing membrane binding of PAR-3 monomers was sufficient to promote segregation. Second, oligomeric PAR-3 generally accumulated to higher levels at the membrane and segregated better compared to monomers or RitC::monomer fusions. At the same time, although oligomer size correlated with membrane:cytoplasmic ratio, consistent with larger oligomers exhibiting better binding to the membrane, we did not observe an increase in segregation efficiency with oligomer size for oligomers ≥2. Such a threshold behavior was also noted by Chang and Dickinson (2022), though for oligomers ≥3. While the root of this difference in threshold is unclear, such threshold-like behavior cannot be readily explained by the observed progressive changes in diffusivity and membrane affinity with increasing oligomer size. It is possible that membrane-association of small oligomers is sufficiently stable that further increases in binding afforded by increased cluster size are irrelevant. Alternatively, oligomerization per se may enable PAR-3 to engage additional mechanisms that further stabilize asymmetry induced by cortical flows, explaining the lack of a clear correlation between oligomer size and segregation efficiency. For example, oligomerization per se could enable positive feedback, which, while presumably not sufficient to drive polarization on its own, could reinforce advection-induced asymmetry to enhance and stabilize polarity (Chang and Dickinson, 2022; Dawes and Munro, 2011; Lang and Munro, 2022). Such feedback would complicate the relationship between oligomer size and segregation efficiency. Thus, a key point of future work will be to determine how, if at all, such mechanisms combine with simple oligomerization-dependent membrane stabilization to enable robust segregation of PAR-3.

In conclusion, here we demonstrate that cortical flows impact a broader range of membrane-associated molecules than previously anticipated and suggest that cortical flows act broadly to shape intracellular protein distributions, including during cell migration, cell polarization, and cytokinesis (Longhini and Glotzer, 2022; Maiuri et al., 2015; Ng et al., 2022 *Preprint*; O’Neill et al., 2018). At least in the case of PAR polarity, this lack of specificity in coupling to cortical flows, combined with a relatively low diffusivity of PAR proteins, requires that the membrane binding of PAR proteins be differentially regulated to achieve efficient and selective transport during polarization.

## Materials and methods

### *C. elegans* strains and culture conditions

*C. elegans* strains were maintained under standard conditions (Brenner, 1974) at 20°C (except where noted) on NGM (nematode growth media) plates seeded with OP50 bacteria. See Table S1 for details of all strains used in this work. Unless otherwise noted, imaging was performed at ∼20°C.

### Strain construction

The insertion of HaloTag into the *par-1* locus was performed via CRISPR/Cas9 based on the published protocol (Dokshin et al., 2018). Briefly, tracrRNA (IDT DNA, 0.5 μl at 100 µM) and designed crRNA(s) for the target (IDT DNA, 2.75 μl at 100 µM) with duplex buffer (IDT DNA, 2.75 μl) were annealed together (5 min, 95°C) and then stored at room temperature until required. PCR products containing the DNA sequence to be inserted, i.e., HaloTag (sequence from Dickinson et al., 2017), and HaloTag with 130 bp homology to the insertion site, were generated and column purified (Qiagen, QIAquick PCR purification kit). PCR products were then mixed in equimolar amounts (2 µg each in a total of 10 μl) and annealed together (heated to 95°C and slowly cooled to room temperature) to generate a pool of products with long single-stranded DNA overhangs to act as the repair template. An injection mix containing Cas9 (IDT DNA, 0.5 μl at 10 mg/ml), annealed crRNA and tracrRNA along with the repair template was incubated at 37°C for 15 min before the debris in the mix was pelleted (15 min, 14,500 rpm). Young gravid N2 adults were injected with the mix and mutants were screened by PCR and sequence verified. A *dpy-10* co-CRISPR strategy was used to facilitate screening (Arribere et al., 2014).

To generate point mutations/small insertions, mutation by CRISPR/Cas9 was performed based on the protocol published by Arribere et al. (2014). To aid screening, silent mutations were engineered into the repair template to introduce a unique restriction site. Briefly, tracrRNA (IDT DNA, 0.5 μl at 100 µM) and crRNA(s; IDT DNA, 2.7 μl at 100 µM) were combined in duplex buffer (IDT DNA, 2.8 μl), annealed together (5 min, 95°C), and stored at room temperature until use. The final injection mix containing Cas9 (IDT DNA, 0.5 ul at 10 mg/ml), annealed crRNA and tracrRNA along with the repair template for the mutation and a co-CRISPR marker (unc-58 or dpy-10) was incubated at 37°C for 15 min before the debris in the mix was pelleted (15 min, 14,500 rpm). Young gravid N2 adults were injected with the mix and mutants were selected, screened by PCR and/or restriction digest, and sequence verified.

Primers and guides used to make all the strains generated in this project can be found in Table S1. Silent restriction sites can be identified in red in the table for the corresponding mutants.

### RNA interference mediated knockdown

RNAi was carried out using the feeding method (Kamath and Ahringer, 2003). RNAi NGM agar plates were prepared by seeding standard nematode growth media agar plates containing 1 mM IPTG (isopropyl β-d-1-thiogalactopyranoside) with 150 μl of an overnight 3 ml culture of bacteria expressing the desired RNAi sequence (which was spiked with 30 μl of 1 M IPTG prior to seeding), and left at room temperature overnight. L4 larvae were placed on seeded RNAi plates for 16–36 h and incubated in a dark box at room temperature, unless stated otherwise.

### HaloTag labeling

JF549-Halo and JF-646-Halo ligands were initially obtained from the laboratory of Dr. Luke Lavis (Grimm et al., 2015) and later purchased from Promega (GA1110, GA1120). Ligands were diluted in acetonitrile into 2 and 0.5 nmol aliquots which were speedvac-ed (dry, 5 min) to remove solvent, and stored at −20°C in a cardboard box. Labeling of the HaloTagged strains was broadly carried out as described (Dickinson et al., 2017) via liquid culture incubation. Briefly, overnight 2 ml OP50-LB cultures were centrifuged, and the pellet was suspended in 200 μl of fresh S-medium solution (150 mM NaCl, 1 g/liter K_2_HPO_4_, 6 g/liter KH_2_PO_4_, 5 mg/liter cholesterol, 10 mM potassium citrate, pH 6.0, 3 mM CaCl_2_, 3 mM MgCl_2_, 65 mM EDTA, 25 mM FeSO_4_, 10 mM MnCl_2_, 10 mM ZnSO_4_, and 1 mM CuSO_4_). The Halo ligand was resuspended in 2 μl DMSO and added to the bacterial suspension to achieve a final concentration of 10 µM. 65 μl of this mixture was pipetted into three different wells in the center of a 96 flat-bottomed well plate and 30–40 L4 worms were picked into each well. Water was added to surrounding wells to keep the plate from dehydration and the plate was covered in aluminum foil and placed in a 20°C shaking incubator overnight. Prior to imaging, worms were pipetted onto unseeded NGM plates. Once the liquid had settled, worms were picked onto new unseeded NGM plates and left for 15 min, and then onto OP50 or relevant RNAi plates.

### Live imaging—cortex and SPT

Embryos were dissected from gravid adult worms (picked as L4’s the night prior to the experiment) using a hypodermic needle into 7.5 μl of Shelton’s Growth Medium (SGM) containing 18.8-µm polystyrene beads (Cat. #18329; Polybead, Polyscience, Inc.). These were mounted between a glass slide and a high precision 1.5H 22 × 22 mm glass coverslip, with the edges sealed with wax (VALAP mixture, 1:1:1, Vaseline, lanolin, and paraffin wax). For worms incubated with Halo ligand, fresh glass coverslips were cleaned (20 min soaking in 96% isopropanol with sonication, three times rinsing with MilliQ H_2_O) and passivated with 30 μl 1 mg/ml PLL-PEG (SuSOS, Cat #PLL(20)-g[3.5]-PEG(2).20 mg) for 40–60 min at room temperature. Prior to imaging, coverslips were rinsed in MilliQ H_2_O, dried on Whatman filter paper, and stored between lens cleaning tissue. Passivation was only effective for 48 h.

Imaging was carried out with a 100× 1.49 NA TIRF objective on a Nikon TiE microscope equipped with an iLas2 TIRF unit (Roper), a custom-made field stop, 488 or 561 fiber-coupled diode lasers (Obis), and an Evolve 512 Delta EMCCD camera (Photometrics), controlled by Metamorph software (Molecular Devices) and configured by Cairn Research. Total internal reflection fluorescence (TIRF) angle was optimized to achieve near-TIRF/HILO as described (Tokunaga et al., 2008). Filter sets were from Chroma: ZT488/561rpc, ZET488/561x, ZET488/561m, ET525/50m, ET630/75m, ET655LP. Images were captured in bright field, GFP/mNG (ex488/ZET488/561m), RFP/mKate/mCherry/Halo (ex561/ZET488/561m).

For advection/diffusion analysis, imaging was carried out at the onset of symmetry breaking. For single molecules (HaloTag), 2,000 × 25 ms frames were streamed alternating between the GFP (NMY-2) and RFP (Halo) channels using a dual-channel emission filter interleaved with 25-ms dummy channels, yielding a characteristic time interval of 100 ms. Bright-field image sequences were captured before and after streams to ensure accurate staging and to monitor embryo viability. For clusters, a multidimensional acquisition was used to sequentially acquire images in the GFP (Clusters), RFP (NMY-2), and bright-field channels with 100 ms exposures and a 1 s time interval.

### Live imaging—midplane

Embryos used for midplane imaging were dissected 8 μl of egg buffer (118 mM NaCl, 48 mM KCl, 2 mM CaCl_2_, 2 mM MgCl_2_, and 25 mM Hepes, pH 7.4) containing 20.8-µm polystyrene beads (Cat. #18329; Polybead, Polyscience, Inc.) between a glass slide and a 1.5 22 mm × 22 mm glass coverslip and sealed with VALAP (1:1:1 Vaseline, lanolin, petroleum jelly). Imaging was carried out with a custom X-Light V1 spinning disk system (CrestOptics, S.p.A.) with 50 µm slits, Nikon TiE with 63× or 100× objectives, 488, 561 fiber-coupled diode lasers (Obis) and Photometrics Evolve 512 Delta EMCCD camera, controlled by MetaMorph software (Molecular Devices) and configured by Cairn Research. Filter sets were from Chroma: ZT488/561rpc, ZET405/488/561/640X, ET535/50m, ET630/75m. Multidimensional acquisition was used to sequentially acquire images in the following channels: DIC, GFP (ex488, ET535/50m), autofluorescence (ex488, ET630/75m), and RFP (ex561, ET630/75m).

To maximize signal to noise for segregation analysis, single images were captured at the end of cortical flow (defined as the departure of the male pronucleus from the posterior cortex) and after nuclear envelope breakdown, typically 5–6 min later.

For FRAP analysis, a line along the cortex was photobleached with a 473 nm laser using an iLAS-targeted illumination system (Roper), and images captured every 5 s.

### Image analysis—single particle detection and tracking

Single molecule and cluster tracking were carried out in Python using the Trackpy package (https://github.com/soft-matter/trackpy). The custom Python code developed for the analysis is available at https://github.com/lhcgeneva/SPT. It implements the Crocker-Grier algorithm to localize particles to subpixel resolution in individual frames by fitting local intensity peaks to a Gaussian point spread function. Detection parameters such as the threshold intensity and diameter of the candidate particles are adjusted empirically for given imaging conditions. Particles are linked frame to frame, with additional user-specified parameters. Parameters were optimized to minimize tracking errors and typically were as follows: feature size = 7 pixels, memory = 0 frames, minimum separation between features = 2 pixels, maximum distance features can move between frames = 4 pixels, minimum track length = 11 frames.

### Image analysis—particle image velocimetry

The local flow field of the acto-myosin cortex was measured by applying particle image velocimetry (PIV) to the NMY-2 image channel using the PIVlab MATLAB plugin (Thielicke and Stamhuis, 2014). Images were bleach corrected and rolling time averaged (2 frames) in Fiji (Schindelin et al., 2012). The posterior region of interest was selected as an ROI, and images were preprocessed with a high-pass filter (size 10), with other preprocessing filters disabled. A FFT phase-space PIV algorithm with two passes (window sizes 64 and 32 pixels) was used with linear window deformation and a 2 × 3 Gaussian point fit. Postprocessing was done with two filters: (1) a velocity filter where manual velocity limits of 0.3 pixels/frame were drawn to remove any outliers, and (2) a standard deviation filter where vectors that more than five standard deviations from the mean in each image were removed. Vectors that were removed were replaced by interpolation. On average, 9.4% of vectors were replaced by interpolation (*n* = 31 embryos). The resulting flow fields have a vector every 16 pixels with additional intervening vectors generated by bicubic interpolation.

### Image analysis—segregation

Raw images were processed using SAIBR using N2 or NWG0038 animals for the autofluorescence calibration as required and cortical concentrations were obtained as described previously (Ng et al., 2022; Rodrigues et al., 2022). Briefly, a 50-pixel-wide (12.8 µm) line following the membrane around the embryo was extracted and computationally straightened. A 20-pixel-wide (5.1 µm) rolling average filter was applied to the straightened image to reduce noise. Intensity profiles perpendicular to the membrane at each position were fit to the sum of a Gaussian component, representing membrane signal, and an error function component, representing cytoplasmic signal. Local membrane concentrations at each position were calculated as the amplitude of the Gaussian component and the anterior- and posterior-most 30% of embryo perimeter averaged.

### Simulations—single particle

Single particle stochastic simulations were carried out to simulate particle movement movies in 2D, and benchmark algorithms relying on detection and tracking of bright fluorescent spots. A custom Python script was used for this purpose and is available at https://github.com/lhcgeneva/SPT. This script creates a series of images with diffusive particles overlayed on top of a noisy background. Spots are blurred by a Gaussian filter to simulate a microscope point spread function.

User-defined variables include the number of particles, diffusivity (in µm^2^/s), a coefficient of variation for diffusivity, time interval between subsequent frames (in seconds), total duration of movie (in seconds), long axis and short axis to define area of each image (in µm).

The updated version of the simulation script for advection analysis also includes the additional user defined variables: dissociation rate (*k*_off_, in s^−1^) and advection velocities perpendicular to the long axis and parallel to the long axis (in µm/s). Default values for each variable for a typical experiment have been defined in the script for easy future use.

When all variables have been defined, the script initializes by assigning particles a lifetime calculated from the probability distribution of the number of particles per frame for a given dissociation rate. The initial position of each particle is assigned as a set of random coordinates within the user defined area. A new position is created for each particle for each frame of its lifetime, by drawing from a probability distribution curve for steps in x and y directions, based on the particles’ defined diffusivity. If an advective component has been defined, the diffusive step is combined with its advective component. When the particle reaches the end of its lifetime, it is given a new random position and considered as a new particle appearing on the membrane. This association rate is equivalent to the defined dissociation rate and allows for the maintenance of a constant number of particles within any given frame. To prevent loss of particles due to boundary conditions, the final area of the images is determined by taking into account the maximum displacement of any particle and extending the image area on all sides by 6 pixels from the position of highest displacement.

### Simulations—partial differential equations

To predict the efficiency of segregation, we modeled advection, diffusion and membrane exchange using a partial differential equation (PDE) model adapted from Goehring et al. (2011b). We used the following governing equations to model a single species:

$$\frac{\partial A}{\partial t}=D\partial_{x}^{2}A+\partial_{x}\left(vA\right)+k_{on}A_{cyt}-k_{off}A$$

$$A_{cyt}=\rho_{A}-\psi \bar{A},$$

where *A* is the membrane concentration, *A*_cyt_ is the (uniform) cytoplasmic concentration, $\bar{A}$ is the average membrane concentration, *ψ* is the surface area to volume ratio, *ρ*_*A*_ is the total amount of protein in the system, *k*_on_ and *k*_off_ are membrane binding and unbinding rates, and *D* is the diffusion coefficient on the membrane. Systems were modeled as 1D membranes 60 µm in length, where x is the position coordinate measured from the anterior pole. The cortical flow velocity (*v*) was specified to match an experimental flow profile as:

$$v=\frac{60-x}{74}e^{-\frac{{\left(60-x\right)}^{2}}{391}}-\frac{x}{1000}e^{-\frac{x^{2}}{100}}\text{.}$$

Values of *k*_off_ and *D* are specified in the main text. For all simulations, we used *ρ*_*A*_ = 1.56 µm^−3^ and *ψ* = 0.174 µm^−1^ (Goehring et al., 2011b), and set *k*_on_ = *k*_off_. Systems were initiated from a uniform equilibrium state and simulated using an adaptive Runge-Kutta scheme (Dormand and Prince, 1980) using custom code written in Python.

To incorporate a mix of short- and long-lived events, we defined a default *k*_off,fast_ = 1 s^−1^, and assigned a fraction of particles to a variable *k*_off,slow_, maintaining all other parameters constant.

### Quantification—advection

Using SPT data, displacements were decomposed into local x and y axes defined as parallel and perpendicular to the local flow vector defined by PIV. For particles within a given bin (e.g., diffusivity or intensity), all individual displacements in x and y over a given time interval, τ, were pooled, plotted as a histogram and fit to a Gaussian distribution with advection (*υ*_*x*_, *υ*_*y*_) given by the mean displacements in x and y, respectively. Coupling coefficients were calculated as the ratio of *υ*_*x*_ to the mean local velocity vector, *ν*. For advection analysis, we let τ = 5 frames (Halo: 5 × 0.1 s, clusters: 5 × 1 s) which provided a reasonable balance between maximizing the ratio of advection to diffusion, which increases with τ, and increasing noise due to the reduction in the number of displacements available, particularly for fast diffusing particles which tend to have shorter track lengths.

### Quantification—diffusion

In general, mean particle diffusivity was calculated from particle step size, where <*d*^2^> is the mean square of all individual displacements for time delay τ, with <*d*^2^> = 4*D*_ss_τ for diffusion in two dimensions. For particles analyzed during the period of cortical flow, only the component perpendicular to the local flow field (∆*y*) was used, with <*d*^2^> = 2*D*_ss_τ. τ = 2 frames (Halo: 2 × 0.1 s, clusters: 2 × 1 s).

### Quantification—segregation

Segregation was defined by several measures. ASI is defined as (A−P)/(A+P), where *A* and *P* are the mean membrane concentrations in the anterior- and posterior-most 30% of the embryo or simulation, respectively. Posterior depletion was defined as *P*/*<I>*, where *P* is as above and <*I*> is the mean membrane intensity. Decay times (*t*_1/2_) in simulations were fit to the values of ASI and posterior depletion from the end of the cortical flow period.

### Quantification—membrane turnover

For FRAP analysis, membrane fluorescence at each timepoint was extracted as described in Image analysis—segregation for the central 30% of the bleach region and a corresponding unbleached region on the opposite side of the AB cell to control for photobleaching during imaging and cell cycle dependent changes in membrane concentration, which were particularly evident for PAR-3. Signal from the bleached region was normalized against the control region and then to pre-bleach values. Note that we used the AB blastomere from two-cell stage embryos for all analysis as they do not undergo polarization and hence exhibit limited spatiotemporal changes in PAR organization during the cell cycle facilitating analysis.

### Statistics

Unless otherwise noted, statistical analysis was performed in Prism 9.0 (GraphPad Software, LLC). All datasets were assessed for normality prior to determination of appropriate statistical test.

### Online supplemental material

Fig. S1 shows rescue of PAR-3(∆69–82) by membrane targeting or oligomerization. Fig. S2 shows FRAP analysis of bulk PAR-3 turnover on the membrane. Fig. S3 shows segregation of PAR-6 under conditions of PAR-3-independent membrane loading. Fig. S4 shows slow-exchanging molecules can dominate the membrane-associated pool of molecules and define the response of the system to cortical flows even if constituting a minority of membrane binding events. Fig. S5 shows oligomerization induces reduced membrane turnover of PAR-2(S241A). Table S1 shows strains and reagents. Video 1 shows monomeric PAR-3 at the cortex undergoes advection. Video 2 shows comparison of advection of Halo::PAR-3 variants. Video 3 shows advection is a general property of PAR proteins.

## Supplementary Material

Table S1 shows strains and reagents.

## Data Availability

Data for all figures are included in the relevant figures or available upon request from the corresponding author. Unless otherwise noted, source code and documentation for simulations and analysis are available at https://github.com/goehringlab.

## References

[bib1] Arata, Y., M. Hiroshima, C.-G. Pack, R. Ramanujam, F. Motegi, K. Nakazato, Y. Shindo, P.W. Wiseman, H. Sawa, T.J. Kobayashi, . 2016. Cortical polarity of the RING protein PAR-2 is maintained by exchange rate kinetics at the cortical-cytoplasmic boundary. Cell Rep. 16:2156–2168. 10.1016/j.celrep.2016.07.047 27524610

[bib2] Arribere, J.A., R.T. Bell, B.X.H. Fu, K.L. Artiles, P.S. Hartman, and A.Z. Fire. 2014. Efficient marker-free recovery of custom genetic modifications with CRISPR/Cas9 in *Caenorhabditis elegans*. Genetics. 198:837–846. 10.1534/genetics.114.169730 25161212 PMC4224173

[bib3] Beers, M., and K. Kemphues. 2006. Depletion of the co-chaperone CDC-37 reveals two modes of PAR-6 cortical association in *C. elegans* embryos. Development. 133:3745–3754. 10.1242/dev.02544 16943281

[bib4] Benton, R., and D. St. Johnston. 2003. A conserved oligomerization domain in drosophila Bazooka/PAR-3 is important for apical localization and epithelial polarity. Curr. Biol. 13:1330–1334. 10.1016/S0960-9822(03)00508-6 12906794

[bib5] Blanchoud, S., C. Busso, F. Naef, and P. Gönczy. 2015. Quantitative analysis and modeling probe polarity establishment in *C. elegans* embryos. Biophys. J. 108:799–809. 10.1016/j.bpj.2014.12.022 25692585 PMC4336357

[bib6] Bray, D., and J.G. White. 1988. Cortical flow in animal cells. Science. 239:883–888. 10.1126/science.3277283 3277283

[bib7] Brenner, S. 1974. The genetics of *Caenorhabditis elegans*. Genetics. 77:71–94. 10.1093/genetics/77.1.71 4366476 PMC1213120

[bib8] Bretscher, M.S. 1996. Getting membrane flow and the cytoskeleton to cooperate in moving cells. Cell. 87:601–606. 10.1016/S0092-8674(00)81380-X 8929529

[bib9] Chang, Y., and D.J. Dickinson. 2022. A particle size threshold governs diffusion and segregation of PAR-3 during cell polarization. Cell Rep. 39:110652. 10.1016/j.celrep.2022.110652 35417695 PMC9093022

[bib10] Costache, V., S. Prigent Garcia, C.N. Plancke, J. Li, S. Begnaud, S.K. Suman, A.-C. Reymann, T. Kim, and F.B. Robin. 2022. Rapid assembly of a polar network architecture drives efficient actomyosin contractility. Cell Rep. 39:110868. 10.1016/j.celrep.2022.110868 35649363 PMC9210446

[bib11] Cuenca, A.A., A. Schetter, D. Aceto, K. Kemphues, and G. Seydoux. 2003. Polarization of the *C. elegans* zygote proceeds via distinct establishment and maintenance phases. Development. 130:1255–1265. 10.1242/dev.00284 12588843 PMC1761648

[bib12] Daniels, B.R., E.M. Perkins, T.M. Dobrowsky, S.X. Sun, and D. Wirtz. 2009. Asymmetric enrichment of PIE-1 in the *Caenorhabditis elegans* zygote mediated by binary counterdiffusion. J. Cell Biol. 184:473–479. 10.1083/jcb.200809077 19221192 PMC2654130

[bib13] Dawes, A.T., and E.M. Munro. 2011. PAR-3 oligomerization may provide an actin-independent mechanism to maintain distinct par protein domains in the early *Caenorhabditis elegans* embryo. Biophys. J. 101:1412–1422. 10.1016/j.bpj.2011.07.030 21943422 PMC3177071

[bib14] Dickinson, D.J., F. Schwager, L. Pintard, M. Gotta, and B. Goldstein. 2017. A single-cell biochemistry approach reveals PAR complex dynamics during cell polarization. Dev. Cell. 42:416–434.e11. 10.1016/j.devcel.2017.07.024 28829947 PMC5575849

[bib15] Dokshin, G.A., K.S. Ghanta, K.M. Piscopo, and C.C. Mello. 2018. Robust genome editing with short single-stranded and long, partially single-stranded DNA donors in *Caenorhabditis elegans*. Genetics. 210:781–787. 10.1534/genetics.118.301532 30213854 PMC6218216

[bib65] Dormand, J.R., and P.J. Prince. 1980. A family of embedded Runge-Kutta formulae. J. Comput. Appl. Math. 6:19–26. 10.1016/0771-050X(80)90013-3

[bib16] Feng, W., H. Wu, L.-N. Chan, and M. Zhang. 2007. The Par-3 NTD adopts a PB1-like structure required for Par-3 oligomerization and membrane localization. EMBO J. 26:2786–2796. 10.1038/sj.emboj.7601702 17476308 PMC1888665

[bib17] Folkmann, A.W., and G. Seydoux. 2019. Spatial regulation of the polarity kinase PAR-1 by parallel inhibitory mechanisms. Development. 146:dev171116. 10.1242/dev.171116 30814118 PMC6451319

[bib18] Goehring, N.W. 2014. PAR polarity: From complexity to design principles. Exp. Cell Res. 328:258–266. 10.1016/j.yexcr.2014.08.009 25128809

[bib19] Goehring, N.W., D. Chowdhury, A.A. Hyman, and S.W. Grill. 2010. FRAP analysis of membrane-associated proteins: Lateral diffusion and membrane-cytoplasmic exchange. Biophys. J. 99:2443–2452. 10.1016/j.bpj.2010.08.033 20959084 PMC2956213

[bib20] Goehring, N.W., C. Hoege, S.W. Grill, and A.A. Hyman. 2011a. PAR proteins diffuse freely across the anterior-posterior boundary in polarized *C. elegans* embryos. J. Cell Biol. 193:583–594. 10.1083/jcb.201011094 21518794 PMC3087016

[bib21] Goehring, N.W., P.K. Trong, J.S. Bois, D. Chowdhury, E.M. Nicola, A.A. Hyman, and S.W. Grill. 2011b. Polarization of PAR proteins by advective triggering of a pattern-forming system. Science. 334:1137–1141. 10.1126/science.1208619 22021673

[bib22] Grimm, J.B., B.P. English, J. Chen, J.P. Slaughter, Z. Zhang, A. Revyakin, R. Patel, J.J. Macklin, D. Normanno, R.H. Singer, . 2015. A general method to improve fluorophores for live-cell and single-molecule microscopy. Nat. Methods. 12:244–250: 3: 250. 10.1038/nmeth.3256 25599551 PMC4344395

[bib23] Gross, P., K.V. Kumar, N.W. Goehring, J.S. Bois, C. Hoege, F. Jülicher, and S.W. Grill. 2019. Guiding self-organized pattern formation in cell polarity establishment. Nat. Phys. 15:293–300. 10.1038/s41567-018-0358-7 31327978 PMC6640039

[bib24] Hao, Y., L. Boyd, and G. Seydoux. 2006. Stabilization of cell polarity by the *C. elegans* RING protein PAR-2. Dev. Cell. 10:199–208. 10.1016/j.devcel.2005.12.015 16459299 PMC1712613

[bib25] Harbury, P.B., T. Zhang, P.S. Kim, and T. Alber. 1993. A switch between two-, three-, and four-stranded coiled coils in GCN4 leucine zipper mutants. Science. 262:1401–1407. 10.1126/science.8248779 8248779

[bib26] Hartman, N.C., J.A. Nye, and J.T. Groves. 2009. Cluster size regulates protein sorting in the immunological synapse. Proc. Natl. Acad. Sci. USA. 106:12729–12734. 10.1073/pnas.0902621106 19622735 PMC2722343

[bib27] Hirani, N., R. Illukkumbura, T. Bland, G. Mathonnet, D. Suhner, A.-C. Reymann, and N.W. Goehring. 2019. Anterior-enriched filopodia create the appearance of asymmetric membrane microdomains in polarizing *C. elegans* zygotes. J. Cell Sci. 132:jcs230714. 10.1242/jcs.230714 31221727 PMC6679585

[bib28] Hoege, C., A.-T. Constantinescu, A. Schwager, N.W. Goehring, P. Kumar, and A.A. Hyman. 2010. LGL can partition the cortex of one-cell *Caenorhabditis elegans* embryos into two domains. Curr. Biol. 20:1296–1303. 10.1016/j.cub.2010.05.061 20579886

[bib29] Holifield, B.F., A. Ishihara, and K. Jacobson. 1990. Comparative behavior of membrane protein–antibody complexes on motile fibroblasts: Implications for a mechanism of capping. J. Cell Biol. 111:2499–2512. 10.1083/jcb.111.6.2499 2277071 PMC2116427

[bib30] Hu, K., L. Ji, K.T. Applegate, G. Danuser, and C.M. Waterman-Storer. 2007. Differential transmission of actin motion within focal adhesions. Science. 315:111–115. 10.1126/science.1135085 17204653

[bib31] Hubatsch, L., F. Peglion, J.D. Reich, N.T.L. Rodrigues, N. Hirani, R. Illukkumbura, and N.W. Goehring. 2019. A cell size threshold limits cell polarity and asymmetric division potential. Nat. Phys. 15:1075–1085. 10.1038/s41567-019-0601-x 31579399 PMC6774796

[bib32] Illukkumbura, R., T. Bland, and N.W. Goehring. 2020. Patterning and polarization of cells by intracellular flows. Curr. Opin. Cell Biol. 62:123–134. 10.1016/j.ceb.2019.10.005 31760155 PMC6968950

[bib33] Jacobson, K., and M. Kapustina. 2019. Going with the flow (or not). Biophys. J. 117:791–792. 10.1016/j.bpj.2019.07.049 31422823 PMC6732662

[bib34] Kamath, R.S., and J. Ahringer. 2003. Genome-wide RNAi screening in *Caenorhabditis elegans*. Methods. 30:313–321. 10.1016/S1046-2023(03)00050-1 12828945

[bib35] Kumfer, K.T., S.J. Cook, J.M. Squirrell, K.W. Eliceiri, N. Peel, K.F. O’Connell, and J.G. White. 2010. CGEF-1 and CHIN-1 regulate CDC-42 activity during asymmetric division in the *Caenorhabditis elegans* embryo. Mol. Biol. Cell. 21:266–277. 10.1091/mbc.e09-01-0060 19923324 PMC2808230

[bib36] Lang, C.F., and E. Munro. 2017. The PAR proteins: From molecular circuits to dynamic self-stabilizing cell polarity. Development. 144:3405–3416. 10.1242/dev.139063 28974638 PMC5665476

[bib37] Lang, C.F., and E.M. Munro. 2022. Oligomerization of peripheral membrane proteins provides tunable control of cell surface polarity. Biophys. J. 121:4543–4559. 10.1016/j.bpj.2022.10.035 36815706 PMC9750853

[bib38] Lee, J., M. Gustafsson, K.-E. Magnusson, and K. Jacobson. 1990. The direction of membrane lipid flow in locomoting polymorphonuclear leukocytes. Science. 247:1229–1233. 10.1126/science.2315695 2315695

[bib39] Li, B., H. Kim, M. Beers, and K. Kemphues. 2010. Different domains of *C. elegans* PAR-3 are required at different times in development. Dev. Biol. 344:745–757. 10.1016/j.ydbio.2010.05.506 20678977 PMC2915941

[bib40] Longhini, K.M., and M. Glotzer. 2022. Aurora A and cortical flows promote polarization and cytokinesis by inducing asymmetric ECT-2 accumulation. Elife. 11:e83992. 10.7554/eLife.83992 36533896 PMC9799973

[bib41] Maiuri, P., J.-F. Rupprecht, S. Wieser, V. Ruprecht, O. Bénichou, N. Carpi, M. Coppey, S. De Beco, N. Gov, C.-P. Heisenberg, . 2015. Actin flows mediate a universal coupling between cell speed and cell persistence. Cell. 161:374–386. 10.1016/j.cell.2015.01.056 25799384

[bib42] Matsuda, S., Y. Kamiya, and M. Yuzaki. 2005. Roles of the N-terminal domain on the function and quaternary structure of the ionotropic glutamate receptor. J. Biol. Chem. 280:20021–20029. 10.1074/jbc.M410513200 15781472

[bib43] Mizuno, K., A. Suzuki, T. Hirose, K. Kitamura, K. Kutsuzawa, M. Futaki, Y. Amano, and S. Ohno. 2003. Self-association of PAR-3-mediated by the conserved N-terminal domain contributes to the development of epithelial tight junctions. J. Biol. Chem. 278:31240–31250. 10.1074/jbc.M303593200 12756256

[bib44] Motegi, F., S. Zonies, Y. Hao, A.A. Cuenca, E. Griffin, and G. Seydoux. 2011. Microtubules induce self-organization of polarized PAR domains in *Caenorhabditis elegans* zygotes. Nat. Cell Biol. 13:1361–1367. 10.1038/ncb2354 21983565 PMC3208083

[bib45] Munro, E., J. Nance, and J.R. Priess. 2004. Cortical flows powered by asymmetrical contraction transport PAR proteins to establish and maintain anterior-posterior polarity in the early *C. elegans* embryo. Dev. Cell. 7:413–424. 10.1016/j.devcel.2004.08.001 15363415

[bib67] Ng, K., T. Bland, N. Hirani, and N.W. Goehring. 2022. An analog sensitive allele permits rapid and reversible chemical inhibition of PKC-3 activity in *C. elegans*. MicroPubl. Biol. 10.17912/micropub.biology.000610 PMC9391946 35996692

[bib46] Ng, K., N. Hirani, T. Bland, J. Borrego-Pinto, and N.W. Goehring. 2022. Cleavage furrow-directed cortical flows bias mechanochemical pathways for PAR polarization in the *C. elegans* germ lineage. bioRxiv. (Preprint posted December 23, 2022). 10.1101/2022.12.22.521633 37729912

[bib47] O’Neill, P.R., J.A. Castillo-Badillo, X. Meshik, V. Kalyanaraman, K. Melgarejo, and N. Gautam. 2018. Membrane flow drives an adhesion-independent amoeboid cell migration mode. Dev. Cell. 46:9–22.e4. 10.1016/j.devcel.2018.05.029 29937389 PMC6048972

[bib48] Onken, B., H. Wiener, M.R. Philips, and E.C. Chang. 2006. Compartmentalized signaling of Ras in fission yeast. Proc. Natl. Acad. Sci. USA. 103:9045–9050. 10.1073/pnas.0603318103 16754851 PMC1482563

[bib49] Padmanabhan, A., H.T. Ong, and R. Zaidel-Bar. 2017. Non-junctional E-cadherin clusters regulate the actomyosin cortex in the *C. elegans* zygote. Curr. Biol. 27:103–112. 10.1016/j.cub.2016.10.032 27989674

[bib50] Raff, M.C., M. Sternberg, and R.B. Taylor. 1970. Immunoglobulin determinants on the surface of mouse lymphoid cells. Nature. 225:553–554. 10.1038/225553a0 4189355

[bib51] Robin, F.B., W.M. McFadden, B. Yao, and E.M. Munro. 2014. Single-molecule analysis of cell surface dynamics in *Caenorhabditis elegans* embryos. Nat. Methods. 11:677–682. 10.1038/nmeth.2928 24727651 PMC4046709

[bib52] Rodrigues, N.T.L., T. Bland, J. Borrego-Pinto, K. Ng, N. Hirani, Y. Gu, S. Foo, and N.W. Goehring. 2022. SAIBR: A simple, platform-independent method for spectral autofluorescence correction. Development:dev.200545. 10.1242/dev.200545 PMC9445497 35713287

[bib53] Rodriguez, J., F. Peglion, J. Martin, L. Hubatsch, J. Reich, N. Hirani, A.G. Gubieda, J. Roffey, A.R. Fernandes, D. St. Johnston, . 2017. aPKC cycles between functionally distinct PAR protein assemblies to drive cell polarity. Dev. Cell. 42:400–415.e9. 10.1016/j.devcel.2017.07.007 28781174 PMC5563072

[bib54] Rose, L., and P. Gönczy. 2014. Polarity establishment, asymmetric division and segregation of fate determinants in early *C. elegans* embryos. WormBook:1–43. 10.1895/wormbook.1.30.2 25548889

[bib55] Sailer, A., A. Anneken, Y. Li, S. Lee, and E. Munro. 2015. Dynamic opposition of clustered proteins stabilizes cortical polarity in the *C. elegans* zygote. Dev. Cell. 35:131–142. 10.1016/j.devcel.2015.09.006 26460948 PMC5963695

[bib56] Schindelin, J., I. Arganda-Carreras, E. Frise, V. Kaynig, M. Longair, T. Pietzsch, S. Preibisch, C. Rueden, S. Saalfeld, B. Schmid, . 2012. Fiji: An open-source platform for biological-image analysis. Nat. Methods. 9:676–682. 10.1038/nmeth.2019.Fiji 22743772 PMC3855844

[bib57] Sheetz, M.P., S. Turney, H. Qian, and E.L. Elson. 1989. Nanometre-level analysis demonstrates that lipid flow does not drive membrane glycoprotein movements. Nature. 340:284–288. 10.1038/340284a0 2747796

[bib58] Tanaka, M., T. Kikuchi, H. Uno, K. Okita, T. Kitanishi-Yumura, and S. Yumura. 2017. Turnover and flow of the cell membrane for cell migration. Sci. Rep. 7:12970. 10.1038/s41598-017-13438-5 29021607 PMC5636814

[bib59] Taylor, R.B., W.P.H. Duffus, M.C. Raff, and S. de Petris. 1971. Redistribution and pinocytosis of lymphocyte surface immunoglobulin molecules induced by anti-immunoglobulin antibody. Nat. New Biol. 233:225–229. 10.1038/newbio233225a0 20480991

[bib60] Tenlen, J.R., J.N. Molk, N. London, B.D. Page, and J.R. Priess. 2008. MEX-5 asymmetry in one-cell *C. elegans* embryos requires PAR-4- and PAR-1-dependent phosphorylation. Development. 135:3665–3675. 10.1242/dev.027060 18842813 PMC13214155

[bib66] Thielicke, W., and E.J. Stamhuis. 2014. PIVlab – Towards user-friendly, affordable and accurate digital particle image velocimetry in MATLAB. J. Open Res. Softw. 2:e30. 10.5334/jors.bl

[bib61] Tokunaga, M., N. Imamoto, and K. Sakata-Sogawa. 2008. Highly inclined thin illumination enables clear single-molecule imaging in cells. Nat. Methods. 5:159–161. 10.1038/nmeth1171 18176568

[bib62] Wang, S.-C., T.Y.F. Low, Y. Nishimura, L. Gole, W. Yu, and F. Motegi. 2017. Cortical forces and CDC-42 control clustering of PAR proteins for *Caenorhabditis elegans* embryonic polarization. Nat. Cell Biol. 19:988–995. 10.1038/ncb3577 28737772

[bib63] Zhang, Y., W. Wang, J. Chen, K. Zhang, F. Gao, B. Gao, S. Zhang, M. Dong, F. Besenbacher, W. Gong, . 2013. Structural insights into the intrinsic self-assembly of Par-3 N-terminal domain. Structure. 21:997–1006. 10.1016/j.str.2013.04.004 23643951

[bib64] Zmurchok, C., and W.R. Holmes. 2022. Biophysical models of PAR cluster transport by cortical flow in *C. elegans* early embryogenesis. Bull. Math. Biol. 84:40. 10.1007/s11538-022-00997-6 35142872

