# Supplementary material for: Design principles for selective polarization of PAR proteins by cortical flows
Source: J Cell Biol. 2023 Jun 2;222(8):e202209111. doi: 10.1083/jcb.202209111 (PMC10238861; doi:10.1083/jcb.202209111)
Supplement: Table S1 — shows strains and reagents. [file JCB_202209111_TableS1.docx]

**Table S1. Strains and reagents**

| **Name** | **Description** | **Source** |
| --- | --- | --- |
| Bacteria |  |  |
| OP50 | *E. coli B, ura-* | Caenorhabditis Genetics Center (CGC) |
| HT115(DE3) | *F-, mcrA, mcrB, IN(rrnD-rrnE)1, rnc14::Tn10(DE3 lysogen: lavUV5 promoter-T7 polymerase).* | CGC |
| *C. elegans* |  |  |
| N2 | Wild type | CGC |
| CGC32 | *umnIs21 [myo-2p::GFP + NeoR, III: 518034 (intergenic)]* | CGC |
| FT248 | *unc-119(ed3) III; xnIs94 [pJN455: hmr-1::HMR-1-GFP::unc-54 3’UTR; unc-119(+)]* | François Robin |
| JJ1473 | *unc-119(ed3) III; zuIs45[nmy-2::NMY-2::GFP + unc-119(+)] V.* | Munro et al., 2004 |
| JK2533 | *qC1 dpy-19(e1259) glp-1(q339)[qIs26] III/eT1 (III;V).* | CGC |
| LP229 | *nmy-2(cp52[nmy-2::mkate2 + LoxP unc-119(+) LoxP]) I; unc-119 (ed3) III* | Dickinson et al. 2017 |
| LP242 | *par-3(cp54[mNG::3xFlag::par-3]) III* | Dickinson et al. 2017 |
| LP620 | *par-3(cp322[HaloTag::par-3]) III (same as LP621)* | Dickinson et al. 2017 |
| LP637 | *par-2(cp329[mNG-C1::par-2]) III* | Dickinson et al. 2017 |
| LP654 | *par-6(cp346[PAR-6::HaloTag]) I* | Dickinson et al. 2017 |
| NWG0062 | *par-2(crk1[par-2(S241A)::gfp]*it328)* | This work |
| NWG0146 | *nmy-2(cp52[nmy-2::mkate2 + LoxP unc-119(+) LoxP])I; unc-119 (ed3) III; zuIs45[nmy-2::NMY-2::GFP + unc-119(+)] V* | This work |
| NWG0147 | *nmy-2(cp52[nmy-2::mkate2 + LoxP unc-119(+) LoxP]) I; unc-119 (ed3) III; unc-119(ed3) III; xnIs94[pJN455: hmr-1::HMR-1-GFP::unc-54 3’UTR; unc-119(+)]* | This work |
| NWG0148 | *unc-119(ed3) III; zuIs45[nmy-2::NMY-2::GFP + unc-119(+)] V; par-3(cp322[HaloTag::PAR-3]) III* | This work |
| NWG0150 | *par-3(it29 [par-3::gfp]) III; nmy-2(cp52[nmy-2::mkate2 +LoxP unc-119(+) LoxP]) I* | This work |
| NWG0256 | *par-3(crk49[HaloTag::PAR-3(∆69-82)*cp322])/hT2[qIs48]; unc-119(ed3) III; zuIs45[nmy-2::NMY-2::GFP + unc-119(+)] V* | This work |
| NWG0271 | *par-3(crk55[HaloTag::PAR-3(∆69-82, GCN4(LI-4mer))*cp322])/hT2[qIs48]; unc-119(ed3) III; zuIs45[nmy-2::NMY-2::GFP + unc-119(+)] V* | This work |
| NWG0291 | *par-3(crk64[mNG::3xFlag::par-3(∆69-82)*cp54])/qC1 dpy-19(e1259) glp-1(q339) III* | This work |
| NWG0295 | *par-3(crk71[mNG::3xFlag::par-3(∆69-82,RitC)*cp54])/qC1 dpy-19(e1259) glp-1(q339) III* | This work |
| NWG0298 | *par-3(crk73[HaloTag::PAR-3([∆69-82,RitC)*cp322])/qC1 dpy-19(e1259) glp-1(q339) III; unc-119(ed3) III zuIs45[nmy-2::NMY-2::GFP + unc-119(+)] V* | This work |
| NWG0309 | *par-3(crk59[mNG::3xFlag::par-3(∆69-82,GCN4(LI-4mer))*cp54]) III* | This work |
| NWG0311 | *unc-119(ed3) III; crkEx1[pNG19: mex-5p::PH(plcδ1)::GBP::mKate::nmy-2UTR + unc-119(+)]; him-5 (e1490) V; par-3(it298 [par-3::gfp]) III* | This work |
| NWG0314 | *unc-119(ed3) III; crkEx1[pNG19: mex-5p::PH(plcδ1)::GBP::mKate::nmy-2UTR + unc-119(+)];*  *him-5 (e1490) V; par-3(it298[par-3(∆69-82)::gfp] )III/hT2[qIs48]* | This work |
| NWG0358 | *par-1(crk94[par-1::HaloTag])* | This work |
| NWG0368 | *par-1(crk94[par-1::HaloTag]); unc-119(ed3) III; zuIs45[nmy-2::NMY-2::GFP + unc-119(+)] V.* | This work |
| NWG0374 | *par-2(crk104[mNG::par-2(S241A)*cp329])* | This work |
| NWG0425 | *par-3(crk138[mNG::3xFlag::par-3(∆69-82, GCN4(IL-2mer))*cp54]) III* | This work |
| NWG0427 | *par-3(crk140[HaloTag::par-3(∆69-82,GCN4(IL-2mer))*cp322]) III* | This work |
| NWG0464 | *par-2(crk1[par-2(S241A)::gfp]*it328) / sC1(s2023) [dpy-1(s2170) umnIs21] III]; crkSi1[pNG19::mex-5p::PH(plcδ1)::GBP::mKate::nmy-2UTR + unc-119(+)]* | This work |
| NWG0465 | *par-2(crk158[mNG::par-2(S241A,GCN4(IL-2mer))*crk104]) / sC1(s2023) [dpy-1(s2170) umnIs21] III* | This work |
| NWG0473 | *par-2(crk159[mNG::par-2(S241A,GCN4(LI-4mer))*crk104]) / sC1(s2023) [dpy-1(s2170) umnIs21] III* | This work |
| UTX32 | *par-2(djd8[Halo::PAR-2])* | Dan Dickinson |
| Recombinant DNA - RNAi Clones |  |  |
| Feeding RNAi: *par-1* | V-9E06 | Source Bioscience (Ahringer Library) |
| Recombinant Nucleotides - CRISPR |  |  |
| sgRNA - *par-1::Hal*o | GCAGTTGCAGCCAATAATGG | IDT DNA |
| Fwd Flanking ODN | ﻿ACCTCATCAGCTGCTCAACC | IDT DNA |
| Rev Flanking ODN | ﻿AGAGGTTTGTGGTCCGACAC | IDT DNA |
| HaloTag fwd ODN | ﻿ATGGCCGAAATCGGAACTGGATT | IDT DNA |
| HaloTag rev ODN | ﻿TCCAGAAATCTCAAGGGTGGAGAG | IDT DNA |
| Fwd ODN for Halo + Homology PCR | ﻿CAACCTTCAACAGGAATCACCGGTACTCGAAAGATTGCTGATCCAAAAGGACGAATTCCGCTCAATTCGACTGCTGTACAAGGACATCGAACTGCAACAGGTGCAGTTGCAGCCAATAATATGGCCGAAATCGGAACTGGATTCC | IDT DNA |
| Rev ODN for Halo +Homology PCR | ﻿GGCTGCAGTACCACCAGCCGCTGGAGTCTTATTGATGAGCTTTGACATCATTGTCGACGAGGTGAGTTGATTCATATACTGCTGCTGCTGAGCATGATCCCGATGACTTGGAATACCACCTCCAGAAATCTCAAGGGTGGAGAG | IDT DNA |
| *par-3(Δ69 - 82)* | ataagcaatattttaatttttcagAGCCAATATCAATCGCTTCCACTGAATGGAAC**CCGC**CGAGTCACTGTTCAATTTGGACGAATGAAAATTGTGGTACCATGGAAGGAATCGgtgagcttttttttcaaatgttcaatcaattttttaatatttttc | IDT DNA |
| *par-3(Δ69 - 82)::RitC* | ataagcaatattttaatttttcagAGCCAATATCAATCGCTTCCACTGAATGGAACTCGCagcgtcgaccacaaaaaaaagtcaaagTGTCCATTTTTTGAGACCT**CCGC**CGCTTACCGTTACTATATCGACGATGTCTTCCATGCCCTCGTGAGGGAGATCCGTCGGAAAGAGAAGGAAGCTGTCCTTGCTATGGAAAAAAAGTCCAAGCCAAAAAATTCGGTCTGGAAGCGTCTTAAGAGCCCATTTCGCAAAAAGAAGGACTCCGTAACTtctggccagggaggatcaggaTGGAAGGAATCGgtgagcttttttttcaaatgttcaatcaattttttaatatttttcttg | IDT DNA |
| *par-3(Δ69 - 82)::gcn4 tetrame*r | cagAGCCAATATCAATCGCTTCCACTGAATGGAAC**CCGC**GGATCCGGTGGTAGAATGAAGCAAATCGAGGATAAGCTTGAGGAAATCCTTTCCAAACTATATCATATTGAGAATGAGCTTGCTCGTATCAAAAAGCTTCTCGGCGAGCGTGGAGGATCAGGATGGAAGGAATCGgtgagcttttttttcaaatgttcaat | IDT DNA |
| *par-3(Δ69 - 82)::gcn4 dimer* | cag﻿AGCCAATATCAATCGCTTCCACTGAATGGAAC**CCGC**GGATCCGGTGGTCGTATGAAACAGCTTGAGGACAAGATTGAGGAATTACTCTCCAAGATTTATCACCTCGAGAACGAGATTGCCCGTCTCAAAAAATTAATCGGTGAGCGTGGAGGATCAGGATGGAAGGAATCGgtgagcttttttttcaaatgttcaat | IDT DNA |
| Fwd screening primer for *par-3* mutants | ATCTTTCATTCACGCTTCAG | IDT DNA |
| Rev screening primer for *par-3* mutants | CTCTCCGAGAAAACGAAAGGGG | IDT DNA |
| sgRNA 1 for *par-3* mutants | GTCCAAATTGAACAGTGACT | IDT DNA |
| sgRNA 2 for *par-3* mutants | GAATGAAAATTGTGGTACCA | IDT DNA |
| *par-2(S241A)* crRNA | CGGTGAAGCGTTCCGAACGT | IDT DNA |
| *par-2(S241A)* HDR ODN | GTGCTCGCCGGCGGAAATTTCCGCCGAAATGGATTCGGCAGGCGTGCCGAACGCTTCA**CTGCAG**CCACCACCAGTGCACAAAGTTTTGCTGAA | IDT DNA |
| Fwd screening primer for *par-2* mutant | TCACCGAGCACATTTGACCA | IDT DNA |
| Rev screening primer for *par-2* mutant | AGCTATTCGGGGCGGAAAAA | IDT DNA |
| *par-2::gcn4 dimer* | ttttcgtttcagatggtccgaaaattgaaaaaccaagagGGATCCGGAGGACGTATGAAACAGCTTGAGGACAAGATTGAGGAATTACTCTCCAAGATTTATCACCTCGAGAACGAGATTGCCCGTCTCAAAAAATTAATCGGTGAGCGTGGTGGATCCGGTaatatcgagagcagctacgacgatttgttcatttgtga | IDT DNA |
| *par-2::gcn4 tetrame*r | ttttcgtttcagatggtccgaaaattgaaaaaccaagagGGATCCGGTGGTAGAATGAAGCAAATCGAGGATAAGCTTGAGGAAATCCTTTCCAAACTATATCATATTGAGAATGAGCTTGCTCGTATCAAAAAGCTTCTCGGCGAGCGTGGAGGATCAGGAaatatcgagagcagctacgacgatttgttcatttgtga | IDT DNA |
| sgRNA 1 to insert GCN4 dimer | AGCTGCTCTCGATATTCTCC | IDT DNA |
| sgRNA 2 to insert GCN4 dimer | GGTCCGAAAATTGAAAAACC | IDT DNA |
| Fwd Screening primer for GCN4 insertion | CATGTGGGCACTCGTACTGT | IDT DNA |
| Rev screening primer for GCN4 insertion | AAACCCGACTTTTGGGGTCA | IDT DNA |
| Key Reagents |  |  |
| JF549-HaloTag | Cat. # GA1110 | Promega |
| JF646-HaloTag | Cat. # GA1120 | Promega |
| Microspheres | Polybead Cat. # 18329 | Polyscience, Inc. |
| Software |  |  |
| Fiji | Image processing | fiji.sc |
| Matlab | Image and data analysis | www.mathworks.com |
| Metamorph 7 | Microscope acquisition software | Molecular Devices |
| Python | Image and data analysis, simulations | www.python.org |
| Prism 9.0 | Statistics and plotting | GraphPad Software, LLC |
